# “Human Beings Is What Women Want to Become, and to Partake of the Garland of Work and Victory.” Visions of Emancipation, Community Spirit, and Social Reform in the First German Women's Movement

**DOI:** 10.3389/fsoc.2019.00064

**Published:** 2019-09-13

**Authors:** Susanne Schötz

**Affiliations:** Chair of Social and Economic History, Dresden University of Technology, Dresden, Germany

**Keywords:** emancipation, women's movement, Germany, Louise Otto-Peters, social reform

## Abstract

My reflections draw attention to the General German Women's Association (German: Allgemeiner Deutscher Frauenverein—ADF), which was founded on 18th October 1865 under the chairwomanship of writer, journalist and publicist Louise Otto-Peters (1819–1865). This event marks the beginning of the organized women's movement in Germany. I will pay special attention to Louise Otto-Peters, who not only initiated the ADF and chaired it for many years, she was also its most important theoretical thinker, and crucially, developed specific policies for women. This happened at a time when women were largely excluded by law from political participation. They did not have the right to vote until 1918 and were therefore unable to play any role in city and state parliaments, or in the Reichstag, either. Until 1908, they were not even allowed to be members of political parties or associations with a political orientation. The first part of this article sets out to depict Louise Otto-Peter's views and visions based mainly on her writings “Das Recht der Frauen auf Erwerb. Blicke auf das Frauenleben der Gegenwart” (The Right of Women to Gainful Employment. Views on Contemporary Women's Lives) from 1866, and “Frauenleben im Deutschen Reich. Erinnerungen aus der Vergangenheit mit Hinweis auf Gegenwart und Zukunft” (Women's Lives in the German Reich. Memories from the Past with Reference to the Present and Future) from 1876. The second part attempts an appraisal of her ideas against the backdrop of the existing gender order and the dominant gender thinking. Part three is interested in the legitimization of her visions and, to this end, examines important—but by no means all—discernible lines of argumentation and interpretations.

“Human beings are what women want to become, and to partake of the garland of work and victory.” With these words, Auguste Schmidt summed up what she had previously set out in a more extensive speech held at the start of the first all-German Women's Conference, which took place on the evening of 15th October 1865 in the hall of the Leipzig Booksellers' Exchange. There, in front of several hundred people, she had spoken about “the natural right of women to raise themselves from their current subordination to the equality they deserve alongside men.” According to Schmidt, this “reformation of a woman's place” or “rebirth of women” would “breathe new life […] into creation.” The women's conference convened on this occasion was “summoned to the service of love for the great women's world in its entirety”; and it was only the inner awareness of the good cause that allowed women not to waver and to enter public life^1^.

How might this type of language—infused as it is with religiosity, and perhaps somewhat pathos- filled, confusing and quite difficult to understand from today's point of view—have affected those present? Did they share the feminist aspiration^2^ toward gender equality formulated here, in which participation in work was apparently intended to play a key role? Why was that? How did those in favor of the idea of equality imagine the new sought-for relationship between the sexes and the new society associated with it? What were the ways and means they considered suitable for moving closer to their goal? And finally: What arguments did they use to promote the idea of equality? Was there a special emphasis on religious convictions here?

These are the questions that have inspired the following reflections. They draw attention to the General German Women's Association (German: *Allgemeiner Deutscher Frauenverein*—*ADF*), which was founded on 18th October 1865, the anniversary of the Battle of the Nations, as a result of the deliberations of the above-mentioned women's conference under the chairwomanship of writer, journalist and publicist Louise Otto-Peters^3^. In most descriptions of the history of the women's movement, this event marks the beginning of the organized women's movement in Germany^4^, despite still remaining relatively under-researched^5^. Most studies on the history of the women's movement as one of the great social and political movements of the nineteenth and twentieth centuries are interested in its heyday, which is understood to be the period from 1894 to the First World War. This period was very much defined by the existence of an umbrella organization of the German women's movement, the Federation of German Women's Associations (*Bund Deutscher Frauenvereine–BDF*), and was characterized by great thematic diversity and differentiation^6^.

As a consequence, we still know too little at present about how the “first generation” of the German women's movement who were active in the ADF understood emancipation, and which social concepts and ideas about gender guided them. Just how the pioneers of the ADF argued against the backdrop of the ruling middle-class gender ideal, and the ideas, values and convictions they drew on are questions that are by no means completely clarified. The fact that religious interpretations of meaning would probably have played a role in the thoughts, feelings and actions of most people at the time, given the still widespread dissemination of religious ideas, has barely been addressed so far, let alone investigated^7^. In general, apart from a few exceptions^8^, religious interpretations only play a role in the relevant depictions of the history of the women's movement where there is no escaping them: In dealing with the large denominational women's associations that originated after the end of the nineteenth century^9^. These were the German Protestant Women's Union of 1899, the Catholic Women's Union of Germany, founded in 1903, and the Jewish Women's Union of 1904, which have also been individually investigated (Kaplan, 1981; Kaufmann, 1988; Baumann, 1992; Breuer, 1998; Wenzel and Wolff, 1999; Maierhof and Wenzel, 2004). However, the subjective significance of religion and church for the people of the nineteenth and twentieth centuries has also only come into sharper focus since the 1990s, following the boom in the social history of religion (Hölscher, 1994; Hübinger, 1994; Blaschke and Kuhlemann, 1996). In this context, aspects of female life have also sometimes been critically examined (cf. in particular, Götz von Olenhusen, 1995; Meiwes, 2000; Bauer, 2006). Nevertheless, it can still be stated that there remain clear shortcomings regarding the investigation of religious orientations among the representatives of the non-denominational women's movement, whether organized in the ADF or in other associations.

These shortcomings, however, are scarcely less evident when researching into secular orientations and into the interactions and mixes of secular and religiously transcendent interpretations among the founders of the first German women's movement who were actively involved in the ADF, and among the men supporting them. At present, I know of about 70 women and 20 men who belonged to this group of people between 1865 and 1890 (cf. Schötz, 2005, p. 153). Biographical research that would allow a closer insight into their ideas of emancipation and the convictions underlying these ideas is only available for a very small number of them.

Louise Otto-Peters, however, is an exception. The research into her life and work, promoted by the Louise Otto-Peters Gesellschaft e. V. over the last two decades, has led to us now knowing more about the intellectual influences that shaped Louise Otto-Peters and the emancipatory ideas she developed^10^. But here too, a great deal of research is still necessary. From her extensive written works, comprising songs, poems, novellas, tales, more than 30 novels, some opera libretti, accounts of history and women's history, biographical portraits, literary criticism, music and theater reviews, and the sociopolitical and women's political journalism published between 1843 and 1895, only a few titles have been taken note of so far and examined in their specific contexts^11^. Most of them still await historical and critical assessment and appraisal. At present, this situation allows neither for a meaningful evaluation of Louise Otto-Peters the writer, journalist and publicist, nor for the politician involved in women's issues, let alone for the ADF as a whole.

In the following, it will therefore only be possible to highlight some key issues and possibly to refer to those still open to research, but not to present a comprehensive analysis of socio-political concepts of women's emancipation and the intellectual influences and values these reflect. I will pay special attention to Louise Otto-Peters, who headed the ADF from 1865 until shortly before her death in 1895. She not only initiated the ADF and chaired it for many years, she was also its most important theoretical thinker and, crucially, developed specific policies for women. This happened at a time when women were largely excluded by law from political participation. They did not have the right to vote until 1918 and were therefore unable to play any role in city and state parliaments, or in the Reichstag, either. Until 1908, they were not even allowed to be members of political parties or associations with a political orientation^12^.

Unlike her fellow campaigners at the head of the ADF, Louise Otto-Peters was able to draw on a wealth of experience in examining the issue of women's rights, as she had already been dealing with it since the “Vormärz” era (the period preceding the March Revolution) and the revolution of 1848/49. For example, she purposefully took over the publication of the association's newspaper “Neue Bahnen” (*New Paths*), first with Jenny Hirsch, and then together with Auguste Schmidt until her own death in 1895^13^. This enabled her to exert a high degree of influence on opinions in the emerging women's movement. Even if the concrete reception of her views within and outside the ADF largely constitutes a desideratum for research, there may well be a great deal to support the view of Ruth-Ellen Boetcher-Joeres that

*“possibly […] the whole women's movement was scarcely imaginable without Otto*—*or more precisely, that it would have developed quite differently without her. Otto influenced everything related to the movement, even its language: She gave terms, such as self-help, rights and duties, freedom, independence that meaning which, from a historical perspective, is so characteristic of the entire middle-class women's movement of the 19th century.”*^14^

The first part of this article sets out to depict Louise Otto-Peter's views and visions based mainly on her writings “Das Recht der Frauen auf Erwerb. Blicke auf das Frauenleben der Gegenwart” (*The Right of Women to Gainful Employment. Views on Contemporary Women's Lives*) from 1866, and “Frauenleben im Deutschen Reich. Erinnerungen aus der Vergangenheit mit Hinweis auf Gegenwart und Zukunft” *(Women's Lives in the German Reich. Memories from the Past with Reference to the Present and Future)* from 1876. The second part attempts an appraisal of her ideas against the backdrop of the existing gender order and the dominant gender thinking^15^. Part three is interested in the legitimization of her visions and, to this end, examines important—but by no means all—discernible lines of argumentation and interpretations. In doing so, I will, as far as possible, trace the genesis of her ideas. Finally, I will return to the questions raised at the beginning by making some observations on them.

## Visions of Emancipation, Community Spirit, and Social Reform

The essay “Das Recht der Frauen auf Erwerb” can be understood as the ADF's programmatic pamphlet. Louise Otto-Peters wrote it in the winter of 1865/66 with the aim of providing a comprehensive account of the motives for founding the ADF, its views and goals, and also of how the first women's conference proceeded. The text also communicated the resolution unanimously adopted by the women's conference, as well as the statute of the association. The idea was to win over “a wider audience” for the endeavors of the newly-created association^16^. This certainly happened in agreement with, if not on the authority of the ADF board.

The second essay from 1876, “Frauenleben im Deutschen Reich,” is somewhat different in character. In the form of this text, Louise Otto-Peters once again, 10 years after the founding of the ADF, presented a major blueprint of her views on women's issues, this time under the new political conditions of the German Empire.

To the best of my present knowledge, this is also her last comprehensive statement on women's emancipation. The book was concerned with tracing the technical, economic and social changes that cultural life had undergone over the previous 50 years, and how these influenced women's lives. It also discusses what further changes she believed would result from this (cf. Otto, 1876). Of particular interest in this context is the last chapter, entitled “Zukunft” (*Future*); here, in the section headed “Zukunftshoffnungen” (*Hopes for the future*), she provided a complex depiction of her vision for the emancipation of women and for social politics. So far, this text has attracted virtually no attention in the research on the history of the first German women's movement. This is astonishing because Louise Otto-Peters, who in this work was not making specific demands in terms of women's politics for the present, but was instead creating an image of women's lives in the future, was able to formulate her own, very personal ideas and convictions without having to be unduly considerate.

This holds true both with regard to board members and to members of the ADF, who may have disagreed on some points, and with regard to the existing association laws for women, since this was not a politically correct association program that was subject to approval, but a draft for the future, a utopian idea. Her chapter “Zukunft,” succinctly introduced with the poem “Drei Jahre, 1865, 1875, 1962,” is an artful device by Louise Otto-Peters, a small political masterstroke even, allowing her to depict her political visions for women and society. However, exactly which visions, understood as images of future events, become clear when examining the above-mentioned texts?

### Work, Education, Emancipation, and Social Reform

The founding documents of the ADF already show the liberation of female work, the betterment of female education and the principle of self-help to be absolutely pivotal points in all efforts to improve the individual and social situation of girls and women. For example, § 1 of the association's statute stipulates: The ADF “is tasked with joining forces in working for the improved education of the female sex and the liberation of female work from all obstacles to its evolvement”^17^. According to § 2 of the program, the appropriate means of remedying this situation were seen to be the

*“agitation by women's education societies and the press, the establishment of productive associations, which are preferably recommended to women, the setting up of industrial exhibitions for women's work products, the founding of industrial schools for girls, the building of girls' hostels, but ultimately also the cultivation of higher scientific education.”*^18^

Thus, the founders of the ADF initially understood “female work” to be the “gainful employment” of women. They regarded promoting employability and opening up the widest possible employment opportunities as an overarching goal “for all” women. In principle, women were to be put in a position of “independence.” According to Louise Otto-Peters, “as linguistic usage suggests, only those who are themselves able to stand—that is, those who are able to sustain themselves on their own two feet and without outside help—can be considered to be independent”^19^. It was therefore her view that helping women to become economically independent by practicing a profession was “the foundation of female independence” and the most important step from which everything else would follow^20^.

However, she did not see the ability to become independent through gainful employment merely as a stopgap measure or as a means of securing one's livelihood against the “vicissitudes of destiny”^21^ in the shape of spinsterhood, widowhood or a husband's employment problems. For her, employment that could secure one's livelihood formed the basis for a life of dignity and self-realization, “as those who are solely dependent on the help of others to advance in the world can never be fully conscious of their own strength, nor achieve the dignity of independence and thus of true humanity”^22^. Just like boys, girls should therefore be trained for a job that corresponds to their abilities and inclinations; daughters as well as sons should be asked by their parents “what they would like to learn and become”^23^. According to Louise Otto-Peters, “they ought to be able to find a field of activity that provides their lives with meaning, secures their livelihood and makes them useful members of human society”^24^. For her, the “main thing” was “to develop the talents slumbering in women, to form characters, to make their lives meaningful and productive for themselves and others”^25^. This is a second, more comprehensive meaning of the term “female work,” which I believe was principally shared by the women around Louise Otto-Peters.

Louise Otto-Peters' concept of female gainful employment does not contain any specific guidelines for or barriers to the development of talents, but its genesis needs further research. In her programmatic pamphlet from 1866, she outlined a wide range of areas for female employment. Starting from the already more or less accessible professions of the artist and the writer, the teacher, the kindergarten teacher, the increasingly more numerous shop girls and saleswomen, the photographers and lithographers, she assumed—due to commercial and technical training beginning to open up for women—they would be employed in the counting houses of merchants, in offices of the railways, in telegraph and post offices and on agricultural estates. In particular, the freedom to pursue a trade which was introduced almost everywhere in 1865, and the abolition of the rights of guilds and fraternities to prohibit individuals from practicing trades caused her to reflect on the independent opening of businesses in trades and crafts by women, presuming they were able to acquire the necessary commercial and technical training. She also considered it possible for women to study medicine and become doctors. She did not even rule out “elevated” factory work for middle-class women, referring to examples from America and Switzerland, as well as to women typesetters who were already working at the time in a Leipzig printing works^26^.

In her “Zukunftshoffnungen” from 1876, she then prophesied^27^ that women would be personally involved in “all concerns that are closest to them,” such as setting up, supervising and running all kinds of institutions for small children, girls' schools, all rescue centers, prisons, hospitals and asylums where female inmates were to be found. Women would also be involved in poor relief, should this still be necessary^28^. There would also be female lawyers and judges who would pass sentence^29^. Her vision is most comprehensive in the area of girls' education. She is confident that at all educational institutions for girls and women, there would be equal numbers of male and female teachers who would be completely equal in terms of pay. She expressly mentions grammar schools and universities as the highest educational institutions to which girls and women would have access, and at which women would teach alongside men. Also, in future, there would be no regulations on how far learning capacity and performance could go, neither for women nor for men^30^. And she continues:

*“Whether or not in this future, women will be called doctors and professors is a matter of complete indifference, just as long as they have had the same opportunity to develop their abilities as men, and the same rights to make use of them. If this is the case, then the goal we have in mind will have been reached.”*^31^

What she put into words here was no longer aimed only at equal participation of women in education and employment, but at the general aspiration to individual self-realization of personal abilities and talents, and the right to use them to contribute to social life. This constitutes a third, all-embracing understanding of “female work” or of work in general. It culminates in the vision of attaining human completeness^32^ through the development and perfection of humanity, which she also refers to as the “rule of all-conquering humanity” or the “ideal of the harmony of humankind”:

*“The goal is the harmony of humankind and this will not be established as long as human beings are still prevented by law or society from achieving harmony with themselves and their environment; and they are prevented from this as long as it is not possible for them—or at least made more difficult by other people—to develop themselves and their abilities, and to use them in their own interest in free self-determination, as well as in the general interest in voluntary subordination and devotion.”*^33^

For Louise Otto-Peters, this vision can be read as an understanding of “emancipation.” Reflected from the perspective of different historical philosophies, the term had been elevated since the 1840s to become a term of movement and purpose. It included liberation from legal, social and political or economic dependencies, the elimination of which would create an empire of freedom without domination^34^. This is exactly what Louise Otto-Peters described in her ideal of the harmony of humankind. Her vision of emancipation applied, of course, to every human being. Accordingly, women's emancipation meant that women should have the same rights and opportunities as men to develop their skills and the same rights and opportunities to employ them. Regarding the women's rights that were to be fought for, she herself used the term “women's question,” thereby distancing herself emphatically and critically from the so-called emancipated of the 1840s (cf. Grass and Koselleck, 1975)^35^.

Her fundamental conviction—that the meaning of human existence is to participate in the perfection of humankind through the development of one's own specific strengths—did not prescribe a direction but applied to all areas of human life. Wherever every man and every woman is willing and able, the development of the individual's own talents for his or her own sake and in the service of the community should be possible—within marriage and the family, in employment, and in public and political life. Her comprehensive vision of emancipation expressly included all women's groups. She wrote:

*“In the future, there is everything to be gained for life in all its parts! The young girl no longer idly dreams away her time; she has her years of learning and preparation, she makes herself useful and only love will lead her to the altar and thus into a new circle of duties which she can also often enough combine with her former profession, just as the man can. The housewife, who can look back on a youth well spent, now turns her house into a temple of satisfaction and beauty, the mother educates her children for the fatherland and humankind and cultivates every ideal in them—the unmarried woman, the widow, the older woman: They all are not unsatisfied; they have a field of activity, either in a profession that feeds them at the same time, or in the community, in political life”*^36^.

The design of a meaningful, useful life that is focused equally on the individual and the common good becomes clear. In it, work in the sense of learning and meaningful activity is pivotal; occupation in the sense of employability and gainful employment unites all groups of women. Gainful employment is also thought of as something that is possible for the married woman, whose duties lie firmly in marriage and family.

In “Zukunftshoffnungen,” the participation of women in public and political life is implied as a matter of course. There is, indeed, no other way within a utopian vision of the free realization of individual abilities and talents: It includes the self-evident right of women to full political participation. Louise Otto-Peters therefore describes a time “when it will simply be considered impossible that one once spoke of the ‘people' […] but understood this to mean only men,” and when one had granted universal suffrage, but allowed half the people, the women, to go without^37^. In the future, whatever the electoral procedure, it would be the same for men and women. It would be worthy of a state based on the principles of the human ideal that women “introduce” their female views and their female will “into law alongside those of the male”^38^.

Alluding to the growing militarism in the German Empire, she refers to a peace balancing on the “tip of the sword” and a people in arms “mostly striving for profit and pleasure” as conditions that do not “correspond to the ideal of the harmony of humankind”^39^. The still prevailing exclusion of one half of the people, the women, from most educational means and civil rights, also shows, according to her, “how little progress humankind has made in its development, especially in the development of humane conditions that benefit all”^40^. For her, such conditions had no claim to permanence, not even to a long duration.

*“Why should we not be able to predict that a time will come when the whole unfortunate and inhuman warmongering with all its barbarity and all its misery will cease? Where the peoples will live peacefully side by side and where any potential disputes […] will be decided by tribunals of nations, but not by the brute law of the jungle?”*^41^

If world history so far had shown “how far or rather how little humankind has advanced in its development, especially in the development of humane conditions that benefit all, without the self- confident and legal participation of women,” why, then, should not even the attempt be made to see how far one could come with it?^42^

Here, the connection between women's emancipation and social reform in Louise Otto-Peters' thinking becomes particularly clear. A society whose ideals are the free development of all its members and the peaceful coexistence of all peoples is unthinkable without the participation of women. It can only be achieved through “the joint work of man and woman, only through the equality of both sexes in all things”^43^. Thus, the participation of women in solving social problems is not only the goal but already a path to it, a path to social reform.

She follows this up with her vision of marriage and family, and the gender relations of the future. It is the image of equal conditions—such as a marriage entered into by completely equal partners and based exclusively on “love and spiritual kinship,” but not on “calculating secondary intentions.” The spouses are not dependent on each other by force of circumstance, but are connected to each other by love and a “common onward striving.” Women will then gladly consider their most important duties to be those undertaken for their families. All in all, however, the fact that women and men not only meet socially, but also get to know each other in the collegial and political intercourse of public life, makes the relationship between the sexes “purer and nobler”; it will no longer be infused with eroticism at every opportunity. The exchange of female and male souls in science and art, politics and religion, or their striving for a common goal will promote mutual spiritual development and make life more noble^44^.

However, Louise Otto-Peters did not equate women's participation with a mere imitation of men's actions^45^. In the future, women would have worked their way through to “a noble femininity”; they would help men in all their endeavors for the good of humankind as “guardians and priestesses of the ideal”^46^.

This idea can already be found in “Das Recht der Frauen auf Erwerb” from 1865, and, indeed, can be traced a long way back. In “Das Recht der Frauen auf Erwerb,” Louise Otto-Peters speaks of the “truly feminine” or the “eternal feminine,” which women must be made aware of, and which must be introduced into humankind, “so that it raises not only the individual but the whole of humankind to higher positions, in order to attain the goal of perfection”^47^. Exactly this could only be achieved if women were not held captive in a small, limited space “in which they atrophy and never learn to know and practice their noblest powers, let alone have the opportunity to make full use of them.” According to Louise Otto-Peters' vision of a specific female role, the woman was therefore “the priestly guardian of the sacred and sanctifying flames of enthusiasm (not only at the domestic hearth but also at the sacrificial altar in the temple of the fatherland), […] without which the whole of humankind is lost!”^48^ While the male sex remained at liberty “to rule the world through physical power and strength as well as through the sharpness of its mind and the stricter logic of its thinking,” the “female sex, precisely on account of its emotional life, its receptivity to everything great and beautiful, its excitable imagination and its aspiring ideal direction” had to be given access “to *co-regency*”^49^.

In view of such an altered, equal female role, it is clear to Louise Otto-Peters that the legal foundations of society have to change, that the civil laws “have to be modified,” based as they are on outmoded beliefs^50^. Gradually, step by step, “not by brutal despotism, but by the mature will of the people,” all barriers that “still restrict women in their independence, in their rights” would be removed—such was her hope^51^. The idea of evolutionary change through reform expressed here finds its complement in Louise Otto-Peters' views on self-help.

### Self-Help, Community Spirit, Social Reform

In her “Zukunftshoffnungen” from 1876, the topic of self-help is not discussed. Given the logic of the depiction, this would also be superfluous, since her ideal of the free development of abilities presupposes individual endeavor and being an active human being.

Instead, the chapter “Selbsthilfe” (*Self-help*) constitutes an important part of her programmatic pamphlet from 1866, “Das Recht der Frauen auf Erwerb.” She introduces this chapter with the words: “Those who do not want to help themselves cannot be helped, indeed they do not even deserve to be helped! *Only what is achieved through one's own endeavors has any value*”^52^. She continues with a thought she had already become convinced of in April 1849, given how women were denied political participation rights and general rights by the revolutionaries. At that time, in the first issue of the “Frauen-Zeitung” (*Women's Newspaper*), which was published by her, she expressed herself in these words:

*“The history of all times and of ours in particular teaches that those who forgot to think of themselves were likewise forgotten—those who did not stand up resolutely for their rights, those who stood idly by, while the others around them continued to work robustly, striding on and on in the service of progress.”*^53^

For Louise Otto-Peters, the notion that female emancipation would only be realized through the interaction of individual and collective self-help of women was, as it were, a quintessence of her entire life up to that point. For her, self-help started with every girl, every woman working “on herself.” To come to the view of oneself as having a purpose, even outside of marriage and family, and therefore having to learn and prepare “to become—not only in an event that might never occur, but in any event—a useful member of human society, and not a burden to anyone”^54^ was what she considered to be the crucial starting point of female self-help. It meant no longer leaving one's fate to chance^55^.

However, since the problems at hand principally concerned the fate of the entire female sex—due to prevailing opinions, existing habits and the legal situation—she understood the necessity of everyone reaching agreement with regard to the most pressing problems and then jointly going into action. In this way, self-help and public spirit merged in her thinking. She was deeply convinced that a real solution to the women's question could only be found “by the women themselves, through their own will and their own strength”^56^. Women would have to decide for themselves what they deemed right and wrong, what should and what should not be done^57^.

She therefore described the founding of the ADF as being of great importance. In her view, its creation supplied the decisive means for promoting common aspirations and safeguarding common interests^58^.

Louise Otto-Peters urged every woman who had so far been longing in vain “for working for the general good” to take the first step and join the ADF: “In doing so, she becomes a link in the great chain of a complete entity, thus joining a community that allows her to be useful to herself and others, and to make her strengths doubly productive in conjunction with those of others”^59^. Throughout her life she emphasized that the ADF had acted under the watchword “All for One and One for All”—as a women's organization based on solidarity and transcending class, implementing the principle of self-help through self-organization^60^.

In purely practical terms, this meant that men could not become members of the ADF. This had nothing to do with misanthropy, but was due to the conviction that women first had to learn “to trust in their own strengths” and “to develop the strength, which to a large extent had until then only been employed in the service of domesticity, into beneficial work for the general good”^61^. For men, it was only possible to obtain honorary membership, bestowed on them by the women. As honorary members, however, they only had an advisory, non-decisive vote, in accordance with section An Appraisal of Louise Otto-Peters' Visions Against the Backdrop of the Existing Gender Order and Dominant Gender Thinking of the association's statute^62^. Consequently, the pioneers of the ADF certainly never excluded cooperation with men as a matter of principle, but they did make it dependent on specific purposes and concerns and, of course, on specific men^63^. This attitude also expressed their commitment to the principles of independence and self-help. Having said this, the above-mentioned section, which virtually turned on its head the prevailing reality of membership being denied to women in most associations, had been the subject of tumultuous arguments, not only during the first German women's conference in 1865, but also at Whitsun 1867 at the first General Assembly of the ADF in Leipzig^64^.

## An appraisal of Louise Otto-Peters' visions against the backdrop of the existing gender order and dominant gender thinking

Louise Otto-Peters' views and visions of emancipation, community spirit and social reform as outlined above stood in stark contrast to the social reality of the nineteenth century, in which women were not entitled to equal rights in any area—neither in marriage and family, nor in education and employment, nor in political life. With a view to the present, much—but by no means everything—has been achieved: According to the prevailing marriage and family law during the lifetime of Louise Otto-Peters, the husband had the final right of decision in all matters. If there was no separation of property, he also had control over his wife's possessions. The introduction of the German Civil Law Code in 1900 did nothing to change this. The partnership of equals outlined by Louise Otto-Peters was only implemented in German law after 1945, and first of all in the GDR. Her vision in the field of education became reality somewhat more quickly, at least in part. Thanks to the great commitment of the German women's movement, the first girls in Berlin were able to pass the German high school exam, the *Abitur*, with special authorization in 1896. From 1908, new rules in Prussia made this possible as a matter of course. Universities began to open up to women in 1900, but we are still a long way from having the same number of female and male professors and equal pay. The world of work had already begun to change considerably during Louise Otto-Peters' lifetime, with the pioneers of the German women's movement making an important contribution through their many initiatives to improve education and training for girls and women. Under current EU law, today there can be no discrimination against women in the form of inaccessible areas in employment, but there are many so-called female and male professions, with a tendency toward lower pay in what are considered to be women's professions. Equal political rights for women and men were achieved in 1918/19; the fact that a woman currently rules in the German Chancellery illustrates the momentous change in the area of political participation. However, we still seem far removed from Louise Otto-Peters' vision of peaceful coexistence of peoples and non-military conflict resolution through international arbitration courts, even though steps in this direction have been taken since 1945 with the creation of the UN and various international treaties.

The concept advocated by Louise Otto-Peters of the “eternal feminine” and its fundamental orientation along a system of two genders have come under criticism. Transgender, intersexuality, and a “third sex,” elusive in terms of its possible expressions, have significantly changed our ideas and convictions. Louise Otto-Peters' vision of emancipation, aimed as it is at the free development of each human being's individual abilities in order to participate in the upward movement and perfection of humankind, is, by contrast, not outdated. Where general human and civil rights are constitutionally enshrined, it has a legal basis, something that is by no means the case everywhere in the world. However, fulfilling the associated aspiration remains a seemingly never-ending task—both individually and collectively.

Just how challenging and explosive, and yet at the same time how innovative the visions of Louise Otto-Peters must have felt in the nineteenth century can only be appreciated if we take into account the middle-class gender and family ideology that was tirelessly propagated at the time.

According to this ideology, it was considered appropriate for women to work in the inner circle of the house—the so-called female profession of wife, housewife and mother—while the outside world of gainful employment, general public life, science and politics was supposed to belong to men^65^. It is true that many things began to change in this respect; artists, writers, kindergarten and school teachers, and governesses gradually became slightly more numerous^66^, and some liberal politicians began to discuss the precarious situation of unmarried middle-class women—the so-called women's question^67^—but fundamentally, little changed in gender thinking.

These middle-class gender concepts were justified by claiming contrasting natural physical and mental characters of the sexes that were derived from the reproductive purpose. Such views were nothing new. They gained fresh significance, however—as Claudia Honegger in particular has shown—with the “rise of the naturalistic sciences” in the second half of the eighteenth and in the nineteenth century, modifying older theological justifications that alleged women were second-rate and inferior by virtue of creation (Honegger, 1991). Doctors and medical philosophers now asserted they were able to document human nature scientifically from the perspective of an “objective view of facts.” Using the method of anatomical comparison, they considered themselves justified in being able to deduce corresponding mental and moral differences from the distinctive physical organization of man and woman—for example, a stronger bone structure, tauter muscles, larger skulls and consequently a stronger intellect, increased vigor and more courage in men; by contrast, a more delicate form, “softer flesh” and thus more emotion, sensitivity, passivity and less self-confidence in women. The different spheres of women and men mentioned above then logically resulted from this, as it were. However, such an order of the sexes, seen as perfectly wise and just, not only represented polar opposites but was also intended hierarchically. Due to their specific physique and psyche, men had the right to the role of head of the family in civil society, as had been the case in the pre-modern era.

Such views were persistent; they did not simply evaporate in the course of time. During the period that is of interest to us, the book “Das Studium und die Ausübung der Medicin durch die Frauen” (*The study and practice of medicine by women*), published in 1872 by physician Theodor von Bischoff, gained some considerable fame, because in it, Bischoff “proved”—scientifically in his view, i.e., on the basis of results of comparative skull and brain anatomy—the fundamental inability of women to study medicine and to work as doctors. He had concluded from, among other things, differences in skull and brain formation, and from smaller sizes of women's brains, the mental inferiority of women. In addition, he considered women to be unsuitable for studying and for the sciences, in particular medicine, on account of their specific female characteristics—he emphasized, for example, timidity, docility, gentleness, weakness of will, being controlled by emotions rather than reason, superficiality and modesty^68^. His book would certainly have been understood by all dissenters as nothing short of a declaration of war. Looking back in 1893, Louise Otto-Peters assessed it as “one of the most crushing blows ever delivered against the female gender in Europe” (cf. Otto-Peters, 1893).

Anyone who stood up for the right of women to self-realization and social participation had no choice but to deal with the ideas of the “master thinkers” who influenced realities in all areas of life. Against this backdrop, how did the pioneers of the ADF argue in order to legitimize their demand for participation? Here, different lines of argument can be discerned, only a few of which I can outline in brief. The focus is once again on Louise Otto-Peters and the essays already mentioned above.

## Argumentations and Interpretations

It is characteristic of Louise Otto-Peters and her fellow campaigners that they initially seemed to take up and affirm the middle-class ideal of marriage entered into out of love, and the purported female profession of wife, housewife and mother; however, they did this only to show that the ideal was in need of modification for a variety of reasons.

### Reality as Argument: Limits to the Feasibility of the Middle-Class Ideal of Gender and Family

One of the most common arguments was to point out the incompatibility or only partial compatibility of the ideal of middle-class women with the actual living conditions of broad sections of the population. Thus, Louise Otto-Peters did indeed describe being a wife and mother as the most beautiful and, to a certain extent, the easiest, “profession of women, as it has simply been sketched out by the hand of nature itself.” That it should be, however, the only profession of women, was, she thought, not only a “confusion of terms,” but also “in glaring contrast to reality”^69^.

On the one hand, she identified this “glaring contrast” with regard to the growing number of manual laborers. In “Das Recht der Frauen auf Erwerb,” she says:

*“Among the proletarians, everyone who does not wish to starve has to work. It is indeed proclaimed always and everywhere: The man is the breadwinner of the family, the acquirer; the woman has only to preserve—but where, as in the lowest classes, the man can often scarcely earn enough to eke out his own existence, then the woman must also take care of hers, and, in turn, the children—boys and girls—must do the same.”*^70^

Accordingly, she considered women's gainful employment, and even child labor, to be indispensable in the lower classes for existential reasons; here, the middle-class ideal of gender and family came up against obvious limits to its feasibility.

She saw the widespread necessity of either earning one's living completely through gainful employment or contributing in some way to the family income, not only for many widows, but also for a considerable number of married women and adult daughters from the middle classes, such as those of civil servants, pastors, lawyers, artists, private scholars and small merchants. In these cases, she observed that widows' pensions, if they were available at all, or the salaries of husbands and fathers were often insufficient to satisfy the needs of families^71^. In her view, the fundamental cause lay in the transformation in the lives of women and families resulting from the technical and socio- economic changes of the preceding decades^72^. In the past, housewives had “much to do, admittedly” and “female helping hands” were welcome in every household in order to be able to satisfy the countless needs involved in keeping house. Things that used to be made at home were now produced by industry, however, and almost all household necessities were purchased for money^73^. Thus, providing for adult daughters in a way befitting one's social station, but also for unmarried female family members who had once had their place in the household of relatives and contributed their labor in one way or another, proved increasingly to be a problem—particularly for members of the educated classes who were employed as civil servants or clerical workers and who were not particularly well paid. It is precisely this question of “where to put all of these individuals who were otherwise occupied in the home”^74^, that had been intensely debated for a time as the “women's question” by liberal politicians since the mid-1860s. Some of them initiated projects to promote the employability of unmarried women from the middle classes in order to provide for female members of their own social group through integration into the labor market in a way that was appropriate to their status and to prevent their social relegation^75^.

But for Louise Otto-Peters, the “women's question” could in social terms not be reduced to lower middle-class women or to middle-class women threatened by social relegation. Throughout her whole life, her thinking included female workers; in fact, it even emanated from their living conditions. As early as 1840, her encounter with the misery of the lace makers and female spinners working in industrial factories in the Ore Mountains had become the key experience in recognizing social ills that particularly affected women and children. Since then, she had been interested in the employment conditions of women doing manual labor and had published a variety of texts on the subject. During the revolution of 1848/49, she demanded that working women not be forgotten when organizing work. This was done in the shape of her famous address of a girl, directed at the Saxon Interior Minister Martin Gotthard Oberländer and the so-called Workers' Commission^76^. When, in 1860, after the long period of repression, she again began to publish openly on women's issues in the “Leipziger Sonntagsblatt,” she focused once more on the question of female gainful employment. This time, she also began to look more closely at the problems of female gainful employment in the “upper classes”; in her series of articles she anticipated much of what she then set out in “Das Recht der Frauen auf Erwerb” in 1865. Among other things, she already addressed the lot of governesses and that of women from the middle classes who, although contributing to the family budget through various types of gainful employment, attempted to conceal this. They wanted to be seen as richer than they were, and considered it “shameful” to work, because this would not be befitting of their “destiny”^77^. She also focused on the examination of the problems of marriages entered into for the purpose of maintenance and of a completely neglected and misguided female education. If this amounted to anything at all, it was to please a man, to be married to and provided for by him, and to be a housewife and mother. However, if for some reason this goal could not be achieved, the situation of women would be a “desperate” one^78^. For this very reason, the main objectives of the ADF were, in view of the few professions that were open to women and especially middle-class women, to advocate opening up new branches of employment for women, but also to work for a change in girls' education toward independence and to boost the creation of education and training opportunities for girls and women of all social classes and strata. Better education and training were intended to open up better employment opportunities and to help in achieving a respected independent position; at the same time, they were supposed to protect against marriages entered into imprudently for the purposes of maintenance and against the drift into prostitution.

### The Special Female Characteristics as a Positive Argument

Arguments for making use of the characteristics that were purportedly specifically female in a way that would benefit female youth or women, and thus be of service to the common good beyond the family, developed plausibility during the course of the nineteenth century. They took up the prevailing discourse of the difference between the sexes, but endowed the female gender characteristics allegedly arising from women's nature with positive connotations, using them to expand women's scope for action in society.

This has been shown to be particularly true in the case of Henriette Goldschmidt, a board member of the ADF^79^. For decades, at the General Assemblies of the ADF, she did indeed put forward many concrete proposals to expand the sphere of activity for women, such as improving the training and social position of primary school teachers, the employment of women in local administrative offices, the establishment of continuing training schools for women, the education of Fröbel-kindergarten teachers, and also the scientific training of female doctors and teachers^80^. Building on the theories of Friedrich Fröbel, who attributed to women a special natural aptitude for the upbringing of children that was allegedly due to their ability to bear children, she made a significant contribution from 1870 onwards to the development of the concept of “spiritual” or “organized motherliness”^81^. At the end of the 1880s, Helene Lange and Mathilde Weber also used this strategy, which was successful in the medium term, in a modified form to justify women's access to the study of high school teaching and medicine^82^.

This connection to the discourse of gender difference, albeit in the version of resulting equivalence and thus equality of both sexes, also becomes evident in the many references Louise Otto-Peters made between 1868 and 1871 to K. F. C. Krause's philosophy and to representatives of Krausism. Krause considered the contrast between man and woman to be the most original, to be “eternal and inalienable”^83^. He assumed specifically male and female “peculiarities,” which he did not, however, rank according to a hierarchical patriarchy, but in terms of equality and complementarity. In his view, men relied primarily on cognition, women on emotion, but both “peculiarities” were of “equal, uniquely characteristic beauty.” “For this reason, man and woman stand on the same level, facing each other as beings of equal worth, not the woman below the man, nor the man below the woman”^84^. Both therefore have completely equal rights—an idea that no other representative of classical German philosophy advocated so resolutely^85^. Krause considered difference linked to simultaneous equality to be the decisive reason why men and women should work together, using these specific abilities in order to create a better, more humane society, just as if they were imperfect halves of a larger, noble whole. Women should therefore not be excluded from any field.

Hermann von Leonhardi, Professor of Philosophy in Prague and perhaps Krause's most important student and follower, had been in personal contact with Louise Otto-Peters and the ADF since 1868. He explicitly invited women as equal participants to the Philosophers' Congress he organized at that time in Prague; in 1869, he then gave a welcoming address at the General Assembly of the ADF in Kassel, in which he emphatically invited women to study Krausism^86^. It is therefore not at all surprising that Louise Otto Peters recommended—in the “Büchertisch” column of the “Neue Bahnen” from 1869—that her readers study Krause, “because this is almost the only philosopher who correctly explains ‘the feminine' and regards women as completely equal to men”^87^. As she stated in a letter in 1869, Krause's assertions as a philosopher were not new to her, but confirmed rather what she herself believed and saw^88^.

“Das Recht der Frauen auf Erwerb” from 1865 does indeed reveal Louise Otto-Peters' very similar way of thinking in the passage where she developed her vision of the “truly feminine” and the “eternal feminine” and the necessity of its development in the service of human perfection. Here, like Krause, Louise Otto-Peters addressed an assumed contrast between male reason and female emotion. She considered “the capacity for enthusiasm, the receptivity for what is great and beautiful, an excitable imagination and a soaring, ideal direction” to be the “eternal feminine.” In 1865, she obviously did not presume a strong love peculiar to women^89^ or a specifically female vision of education or caring, as supported by other protagonists of the women's movement; such thinking is also not discernable in the “Frauenleben im Deutschen Reich” from 1876. Quite the contrary: In “Das Recht der Frauen auf Erwerb,” she remarked that there were certainly many girls “who carry within them different skills and aspirations other than to occupy themselves with children,” and asked why they should be forced into something they did not care for^90^. For her, the “eternal feminine” needed to be introduced into the development of various specific talents, as something which had been granted to women by the Creator, “as divine legacy”^91^. Religious and philosophical interpretations thus complemented each other as far as she was concerned.

I consider Louise Otto-Peters' concept of the “eternal feminine,” in contrast to other interpretations, to be part of her comprehensive vision of emancipation, in which women have the right to use their own specific nature to perfect not only men but all of humankind—just as men must also employ what is specific to them. With this view, she reacts to the dominant middle-class notion of the difference between men and women, not only physically but also psychologically, and in particular creates a connection to the different categories of reason and emotion. However, “the capacity for enthusiasm, the receptivity for what is great and beautiful, an excitable imagination and a soaring, ideal direction” emphasized by her are qualities that can in principle be used in every area of private, social and political life. They thus support her comprehensive vision of emancipation as the free development of one's own strengths in the service of humanity, without restricting women in any way. This concept neither views femininity as superior in working to ennoble humankind^92^, nor does it assume femininity to be a primary source for altruism, compassion and love based on the biological capacity for motherhood, in this way declaring the female side to be better and more humane, as later theorists of the women's movement, such as Helene Lange did (cf. Greven-Aschoff, 1981). It is therefore difficult to describe Louise Otto-Peters' idea of the “eternal feminine” as an important starting point for the theoretical framework of difference and dualism of the sexes under the terminology of the “cultural task of women” in the later German women's movement (cf. Greven-Aschoff, 1981, p. 43). It would be a misinterpretation to have understood it in this way.

To date, we do not know how the concept of the “eternal feminine” was received by her fellow campaigners on the board of the ADF, whether it was of any significance, or whether Henriette Goldschmidt and Louise Otto-Peters ever discussed their different ideas of femininity. This is potentially a wide field of research—if other influential activists of the emerging women's movement inside and outside the ADF are also included in the investigation of femininity and gender concepts.

It is also a largely open question when and under what influences Louise Otto-Peters developed the idea of the “eternal feminine.” Her essay “Das Ewig-Weibliche” (*The Eternal Feminine*) in the “Frauen-Zeitung” from 1851, oft-quoted in this context, represents more of an endpoint or the perfected product; it is also not a moderate answer to the question of “How to move forward?” after the failure of “radical activity” by women in the revolution of 1848/49 to lead to an improvement of their legal situation^93^. It would be a worthwhile task to trace the genesis of her understanding of gender roles and the construct of the “eternal feminine” in the 1840s on the basis of autobiographical writings, manuscripts from her estate, journalistic works and literary output. As Gisela Bock showed, thoughts about the “female sphere” or “mission” had been circulating in Europe since the 1830s. These legitimized the opposite of domesticity—namely female occupation or other activities outside the family. It remains to be examined whether Louise Otto knew the bestsellers by Sarah Lewis, Louis-Aime Martin, Marion Kirkland-Reid or Juliette Adam-Lamber (cf. Bock, 2000). According to autobiographical recollections from 1871, it was her later husband, August Peters, who, on the occasion of them meeting in person in January 1849, put into words for the first time what she “probably already thought, but did not yet dare to say publicly”: That the “eternal feminine” needed to be brought to fruition in humankind, since only in this way would true progress toward the goal of humanity be possible” (cf. Otto, 1871). So, did she adopt the term of the “eternal feminine,” first appearing at the end of Goethe's Faust II, from August Peters?

### The Discourse on Human Rights and Humanity

Research to date on the history of women's movements has highlighted their links to the principles of the Enlightenment and Liberalism^94^. According to Ute Gerhard, this history has, since 1789, always been about the same contradiction—about the promise or the conceivability of freedom and equality for women and about women's rights not being honored or being insufficiently implemented^95^.

In fact, the sources demonstrate a connection to natural law and to the discourse on human rights and humanity of the Enlightenment and of Classicism among many women who spoke up within the ADF—but also among the men who supported its endeavors. They have in common the fundamental conviction of the complete and natural equality of women and men as “species-beings.” In particular, the idea of the freedom of the individual—inalienably inherent in every human being due to them having been endowed with reason, and understood as the necessary basis for their self-development—and the ideas of human dignity and legal equality, which were to enable every human being to improve their social position through talent, diligence and good fortune^96^, offered great potential for justifying claims to women's emancipation. Referring back to the ideas of the Enlightenment, those debating in the ADF repeatedly pointed out the political immaturity and dependency imposed on women as well as the absence of their rights. In accordance with the aims and objectives of civil society, they therefore advocated granting equal rights to both sexes, because if in the name of human rights, people were fighting against a corporate society based on birth and descent, and if formal equality in all rights was one of the fundamental principles of the new order, then the legal inequality of women and men would legitimately also have to be eliminated. In principle, most ADF members probably wanted this to include political participation rights, which, however, were not legally enforceable in Germany for the time being.

In Auguste Schmidt's speech at the ADF's founding conference, references not only to religion but also to the Enlightenment and natural laws had already been touched upon, when she had developed “in a longer speech, the *natural entitlement of women* to raise themselves up from their current subordination to the equality due to them alongside men”^97^. It is precisely this aspiration to promote women's natural entitlement to full equality with men that led Louise Otto-Peters to write “Das Recht der Frauen auf Erwerb” as the programmatic basis for the ADF. As we have seen, she legitimized her opinions and ideas with a wide variety of arguments. However, it becomes clear that she borrowed directly from the human rights discourse of the Enlightenment, particularly when giving her reasons for the principle of independence and self-help. For example, she wrote the following sentence as a comment on her conviction that a real solution to the issue of women's rights could only be found by the women themselves, through their own will and their own strength: “The *right to free self-determination* is the holiest and most inalienable of every being endowed with reason”^98^. This “most simple right to human dignity” was not to be denied to women by anybody,

*“and where it should be tried, they must resist with all the consciousness of their moral dignity until the victory of humanity finally becomes a general victory”*^99^.

Time and again, she expressed the basic conviction that it was the task of the ADF

*“to broaden the sphere of action for women—not only unilaterally, but regarding all the talents given to them and the unlimited assertion of these in all circumstances of life, in the family, the community, the nation, and in the whole of humankind.”*^100^

At the General Assembly in Eisenach in 1872, she argued that the ADF strove “to achieve what is every being's inalienable right: The free development of their talents and strengths”^101^. As we shall see, in “Das Recht der Frauen auf Erwerb,” she described the right to free self-realization as a right granted to every creature by the Creator^102^. So, once again, Louise Otto-Peters' philosophical and religious interpretations complemented each other.

The discourse on human rights and humanity of the Enlightenment and Classicism can be understood as a common bond of the values aspired to by the ADF pioneers; as a bond that united them across all other differences. In 1868, Louise Otto-Peters put it as follows:

*“In our striving, we women […] take the view of pure humanity, or if you will, of natural law; when it is a question of coming closer to our goals: A dignified existence for everyone, including for women, we do not ask for any philosophical or religious, political, national or social declaration of belief” (Otto-Peters*, *1868**)*.

This commitment to participatory rights for women and to humanity obviously had a unifying effect, despite differences in the understanding of femininity; otherwise, the decades of collaboration between Louise Otto-Peters and Henriette Goldschmidt on the board of the ADF, for example, would not have been possible.

Louise Otto-Peters herself had already been heavily politicized under the conditions of the unfolding German national movement in the “Vormärz” era. Political convictions that were based on the ideas of the Enlightenment and that manifested themselves in the growing liberal movement—but even more so in the democratic movement which, to a larger extent, factored in the social question of the working classes—attracted her greatly. Seen from the perspective of their historical genesis, such political convictions were arguably the first to develop significance for the formation of her feminist emancipatory thinking. However, under the influence of the reformed religious interpretations of German Catholicism, these would have complemented each other symbiotically very early on, probably no later than 1845.

As Louise Otto-Peters later pointed out repeatedly, her political interest had already been awakened in her parental home, where her father regularly read the newspaper to his daughters and now and again discussed political events with them. Reading classical literature together—works by the Romantics and the “Junges Deutschland”—will probably also have contributed to arousing a general political interest. Before she could even read, she knew entire ballads and poems by Friedrich Schiller by heart, and she later emphasized how important his female characters and his liberal texts that were directed against prejudice had been for her (cf. Hundt, 2004).

In her essay “Die Theilnahme der weiblichen Welt am Staatsleben” (*The participation of the female world in political life*), published by Robert Blum in the “Volkstaschenbuch Vorwärts” from 1847, she identified the general sources of the emerging political interest of women in the 1840s: First political poetry, in particular the young German poets, then the political conflicts in the state parliaments, to which women were admitted as spectators in Saxony, and finally the religious movement of German Catholicism. All this would probably have applied to herself as well—alongside the autodidactic studies I consider to be eminently important (cf. Hundt, 2004). In this process, her friendly relationships with Robert Blum and Ernst Keil played an important role for the young Louise Otto at the beginning of her literary career, with her acquaintance and friendship with socially critical Austrian poets being of particular significance (cf. Hundt, 2006). Following conflicts with Austrian censorship and police authorities, the young Austrians had moved to Leipzig—Saxony's book, publishing and trade fair city. Among them was Karl Beck, one of the most popular German- speaking political poets of that time. As early as 1840, Louise Otto was inspired by the ideas of equality and freedom he proclaimed, by the denunciation of social destitution, the hope for forthcoming political reforms and his passion for modern technology (cf. Hundt, 2006, p. 120). Karl Herloßsohn—in whose “Komet” she published—also belonged to this circle, as did Hermann Rollett, whom she described as a personal friend and political kindred spirit. In 1848, Rollett published a “Republikanisches Liederbuch” (*Republican Songbook*) in several editions. He was a well-known democrat who was persecuted after the suppressed revolution of 1848/49, and later belonged to the contributors to her “Frauen-Zeitung.” Eduard Mautner and Alfred Meißner also counted among their number. She esteemed the latter more highly as a poet of liberation than Robert Prutz, Georg Herwegh, or Ferdinand Freiligrath (Hundt, 2006). Women were also granted certain rights of participation in the politically lively literary scene of the “Vormärz” era. At any rate, the democrat Robert Blum asked in his “Sächsische Vaterlandsblätter” of 1843 how women would have to express their participation in political life if everyone were called upon to participate in the community and in political life. The reader Otto replied to his question and began a journalistic career under her own name with a series of articles on the topic: It is this series of articles that is regarded by some female historians as the beginning of the history of the German women's movement^103^.

In these pages, it is not possible to trace her own political actions and the exact manner in which her understanding of political participation took shape from the 1840s onwards, through the revolution of 1848/49 and on to the ADF period. Her courage during the 1848/49 revolution remains outstanding: She intervened in the political events, in particular with the already mentioned “Adresse eines Mädchens” and with the publication of the “Frauen-Zeitung” under the slogan “I Recruit Female Citizens for the Realm of Freedom.” Her understanding of democracy was significantly broader than that of most democrats: For example, in the journal “Sociale Reform,” published by Luise Dittmar, Louise Otto-Peters spoke out in favor of women's voting rights as early as 1848^104^. The “Frauen-Zeitung” as a medium for articulating women's interests was founded by her partly on account of the disappointment she felt at the circumstance that revolutionaries who otherwise acted liberally and democratically had forgotten to demand women's rights in 1848/49. A feminist perspective resulting from a comprehensive understanding of freedom and democratic rights was characteristic of her.

The report published by Julius Mühlfeld in the “Mitteldeutsche Volks-Zeitung” on the founding of the ADF shows that it fell within the context of the human rights discourse of the Enlightenment and even of fundamental liberal and democratic values, and was understood by contemporaries in exactly that way. As a longstanding personal friend of Louise Otto-Peters and a supporter of women's emancipation, he concluded his report almost imploringly with the following words:

*“Our sincere wishes and sympathies […] accompany the beautiful and great work that has begun. Themselves unworthy of freedom are those who fight against the striving for freedom, and contemptuous are those who, in the service of prejudice and egotism, despise and suppress the rights of others to free human dignity, free work and self-determination. May the last opponents of the true, worthy emancipation of women towards spiritual and social independence alongside men, which is necessary for the welfare and salvation of humankind and of future generations, not forget this!”*^105^

This is an expression of the knowledge concerning the widespread opposition toward the emerging women's movement, to be expected not only among conservatives, but also among liberals and democrats. Even in 1865, there was only a very small minority among democrats who included women in the human rights program of the Enlightenment.

Arguably, the pioneers of the ADF, in calling for universal human rights for women, were on a belated path of catching up and were now discovering for themselves ideas that had already preoccupied men for decades. This seems problematic to me, however. As far as we know, women were not included in the thinking about general natural rights. With few exceptions—one being K. C.

F. Krause—the great master thinkers, such as Rousseau, Campe, Kant, or Fichte, when they philosophized in general about human destiny, contemplated male destiny, while at the same time always emphasizing the purported natural profession of wife, housewife and mother for women. It is therefore more apt to phrase it this way: The pioneers of the women's movement interpreted the texts of the philosophers anew. Completely in keeping with Immanuel Kant's understanding of the Enlightenment, they had the courage to use their own intellect and to overcome the state of immaturity and heteronomy. With the deepest conviction, they claimed for themselves the general human rights as these had been set down. Auguste Schmidt phrased it very aptly on the inauguration evening of the first German women's conference in Leipzig in 1865: “Human beings is what women want to become, and partake of the garland of work and victory.”

## Religious Interpretations

With regard to Louise Otto-Peters' visions of the emancipation of women, religious interpretations have scarcely been addressed so far^106^. This stands in clear contrast to the existence of—or even saturation with—such visions in some parts of her more comprehensive writings on women's emancipation. The same is also true of her 1865 programmatic essay “Das Recht der Frauen auf Erwerb,” to which the most frequent assessments of Louise Otto-Peters' positions on women's politics in the research on the history of the women's movement refer. So far, religious legitimizations of the claim to equality and emancipation have manifestly been overlooked—including by me—because they have not been the subject of investigation. The hitherto identified influences on Louise Otto-Peters' thinking, in particular the human rights discourse of the Enlightenment, but also the way she took up the discourse of the postulations of diversity, seemed to give satisfactory explanations in accordance with individual expectations. However, this does not do sufficient justice to the historical protagonists: Louise Otto-Peters also demonstrably argued as a Protestant Christian.

In “Das Recht der Frauen auf Erwerb,” she made the case for the principle of self-help—so fundamental to her—with reference to the highest authority, God's will:

*“Only their own strength can ennoble and exalt human beings, that strength whose development and consolidation is the will of God, who created all beings so that they would develop all the abilities that lie dormant within them and strive for free development and moral perfection.”*^107^

In her view, those who persisted in inertia and apathy, without making an effort, sinned not only against their fellow human beings, who looked after them without their deserving it, but even more so against God^108^. For Louise Otto-Peters, the active human striving for perfection is the human willed by God. This self-evidently applies to men as well as women, with their specific intrinsic characteristics.

In order to lend plausibility to her idea of the unfolding of the “eternal feminine” in the service of the development of humankind, she dealt in detail with fears that exactly those most beautiful female qualities might be lost if girls and women were educated to become more independent and to participate in political life. In defense of her idea, Louise Otto-Peters argued that the Creator granted every creature the right to allow themselves space and freedom for the innate peculiarity of their being to develop fully; now it was only this particular right that “women claimed and had to claim if they did not want to fail in the purpose of the Creator”^109^. And she continues:

*“Man and woman have emerged from the hands of the divinity or of creation […] as two equal creatures; but the dissimilarity of their characteristics also holds true in the life of the soul. Balancing out this dissimilarity happens in the union of both. The man as such and the woman as such are entities of equal significance; only when both are united do they form a whole. This is how the wisdom of creation, which subordinates none to the other, wished it.”*^110^

The similarity of these thoughts with those of the philosopher Krause is astounding—was it possible that Louise Otto-Peters already know his writings or those of his students before 1868, after all? And since the wording in the 1851 essay on the “eternal feminine” is almost identical, did she already know Krause's philosophy at that time?

In 1865, however, she invoked the highest possible authority by referring to none other than God, who had equipped human beings as men and women differently but equal, and therefore possessing equal value, while from 1868, she quoted the philosopher Krause as the scientific authority. In “Das Recht der Frauen auf Erwerb” she continues:

“What has been bequeathed to woman by the divinity, to bring to bear in his power and holiness against the predominance of either a cold or brutal force, should not be a futile endeavor in the general development.”

In her view, it was precisely this “eternal feminine” that had to be brought to the consciousness of women and to the fore in humankind in order to attain perfection^111^. In order for women and men “to participate in the work of the century, together with one another in dignified union within marriage and *alongside* one another outside of it,” the ADF called for a changed and independent position of the female sex^112^.

What is interesting about these arguments is that they completely disregard—as if they did not exist—the interpretations of the story of Creation that had been customary for many centuries, and according to which the secondary status of the female sex resulted either from the later creation of Eve from Adam's rib or from Eve's sin^113^. Obviously, in the above case, another concept of faith was relevant for Louise Otto-Peters—that of the holiness of the dissimilarity and equality bestowed by the Creator upon man and woman. Lucian Hölscher describes this adjustment of religious beliefs to the needs of place and time as a culture of reflection typical of Protestant middle-class religiosity in the nineteenth century^114^. From the point of view of the history of ideas, Hegel's interpretation of the Reformation had made a decisive contribution to this. According to him, the freedom of the subject had entered the world with the principle of Protestantism, for the authority of the Church was replaced by the Bible, from which everyone was supposed to teach themselves and define their consciences^115^. Faith could only be substantiated in the subject's self-reflection and, in view of the challenges of life, also had to be examined constantly for the religious truths that were of personal significance. Did Louise Otto-Peters know such considerations from her Hegel studies? Or had she been brought up in this spirit during her religious instruction as a child and adolescent? There is a lot to be said for this, because her autodidactic studies in the years 1841–1843 illustrate the fact that she applied herself extremely seriously to gaining knowledge^116^. At that time, she was particularly interested in the interrelations between religion and philosophy, religion and nature, and between God and a thinking human being^117^. However, it is difficult to say when religious convictions became significant for Louise Otto-Peters' thoughts on women's emancipation. As Johanna Ludwig shows, Louise Otto-Peters attached great importance—in later autobiographical statements—to her confirmation and in particular to her confirmation quote. She had been blessed with the Bible saying: “Be faithful unto death, and I will give you the crown of eternal life.” Louise Otto-Peters subsequently described this scene several times, always emphasizing how moved she had been and how much she accepted these words as the maxim of her life. In 1869, in “Genius des Hauses” she stated:

*“Sublime shivers came over me at that moment—life lay before me as a great, wide arena and I prayed full of fiery devotion, although not that I would be spared the fight, but precisely that it might come—come with all its force, so that I might then prove myself to be a worthy fighter, so that I might really deserve a crown of victory. Here, before the throne of God, everything was indeed equal, whether man or woman—even the trembling girl's hand was allowed to take up the sword and wield it in this fight*.”^118^

According to this portrayal, the 15-years-old Louise Otto-Peters, on account of her religious education and upbringing, already understood herself to be equal with men before God. She therefore laid claim to a life of struggle and confrontation to justify God's trust in her and prove herself to be worthy of it. It is difficult to determine to what extent this depiction was a subsequent stylization in order to show how strongly her conviction of men and women having equal worth and equal rights was based on her faith from her earliest youth, with faith itself therefore being the source of her motivation. Presumably, as a confirmand, she was convinced of the fundamental capacity for salvation of all human beings and of their duty to prove themselves worthy to God, to do good and to strive for their own perfection. However, it is unlikely that she recognized in this a potential for women's emancipation or that she was, in 1834, interested in the issue of women's rights.

In my opinion, her basic religious conviction that man and woman were equal before God was conveyed to her not through the Protestant teachings of her childhood and youth, but only later through German Catholicism. Her autobiographical studies and her increasing political interest since 1840, influenced especially by political poetry, will also have contributed to this. In 1847, she wrote in the “Volkstaschenbuch Vorwärts,” published by Robert Blum: “German Catholicism gave us the shibboleth of a general spiritual equality before God, of priests and of laypeople, scholars and the ignorant, men and women” (cf. Otto, 1847). But in 1845, she had already given an enthusiastic account in the “Wandelstern” newspaper of a sermon by Johannes Ronge in Dresden. The founder of German Catholicism was on a journey across Germany to promote his cause at that time. Having witnessed Ronge's sermon, she described it as the “most solemn hour” of her life, as an hour that had been “a piece taken from world history”^119^.

There were three thoughts in particular that she emphasized: Firstly, Ronge had described it as the task of the nineteenth-century Reformation to fulfill Christianity, because the Reformation had made no more progress for three centuries. The Reformation would recognize the moral world order and human destiny and would reintroduce them into world history. Both were based on these words of Christ: “Become perfect as your heavenly Father is perfect, and love one another.”^120^ Secondly, according to Ronge, women also demanded their part in the struggle of world history:

*“And so it shall be, and so women, too, shall help in their way to build our holy work, and shall not be left behind where we are called upon to work for the people and the fatherland and the holiest human rights.”*^121^

In Ronge's view, German Catholicism as the “faith of freedom and love” created “a priestly people entirely made up of high priests and high priestesses.” But it was not only the thoughts of women participating with equal rights in this religious renewal movement and the right of every human being, including women, to perfection that were extremely important for Louise Otto's own thinking. In her account of his appearance in Dresden, she also emphasized a third fundamental idea of Ronge's—his reply to the accusation that the new movement took too much notice of earthly life: Christ, who healed the sick and fed the poor, Ronge said, had not wanted “millions to be cast into servitude and misery and to have their human rights taken away, and in return have heaven opened up to them.” According to Ronge, the German Catholics taught love for people as Christ had done, they were united in their love for him, as one Christ^122^. This thought, then, meant religious authorization for a program of inner-worldly living, directed at common action in solidarity against the ills of the present and for human rights. It was thus oriented decisively toward the common good and had its place in the democratic movement.

If all three thoughts are merged from the perspective of women's emancipation, the result is a participation of women in the improvement of the world that is legitimized by religious ideas and a right for women—likewise legitimized by religious ideas—to partake of human rights, the first of which was the right to personal self-realization toward perfection. It was precisely this program that determined the visions of women's emancipation developed by Louise Otto-Peters in her essays “Das Recht der Frauen auf Erwerb” and “Frauenleben im Deutschen Reich.” It determined even more, however: Her “Zukunftshoffnungen” from 1876, which included not only women but all human beings.

As she stated in an article in 1848, Louise Otto-Peters developed, in an explicit departure from the “Orthodox Christian standpoint,” a Christian position “where Christianity and humanity are equally important”^123^. This was obviously possible within the framework of her church, because she did not become a German Catholic, despite her being very close to the religion and having many close acquaintances in her personal environment^124^. The program of the “Frauen-Zeitung,” published by her in 1849, reveals religious as well as political and socio-economic interpretations influenced by the religiosity of German Catholicism; but the detailed depiction of these interpretations would go beyond the scope of this article^125^. However, already at that time, she stressed that “true freedom” was indivisible—that is, that political, social and religious freedom can only mean real freedom as a unity and that the civil rights and liberties for men are only rights of one half of humankind (cf. Otto, 1849b). Here, too, political, social and religious interpretations were already intertwined.

After a visit to Louise Otto-Peters in 1869, Paul Hohlfeld, a follower of Krause, wrote in a letter to Hermann von Leonhardi that judging by her religious point of view she was a rationalist^126^. This probably captures it well: Various characteristics—emphasized by Lucian Hölscher—of a religiosity determined by the rationalism of the Enlightenment are reflected in her visions, such as the belief in a general world plan of improvement and perfection, which included the belief in the perfectibility of human beings and in inner-worldly cultural, moral and scientific progress; the belief in the divine principle of reason and in the discernibility of God's hand in nature and history; the idea of God as a loving Father who ultimately accepts all his children; feelings of humility and gratitude toward God and feelings of love and humanity toward humankind, from which a wide field of activity arose^127^. However, inasmuch as Louise Otto-Peters' religiosity expressly included the belief in completely equal rights given to women by God, it would be even more appropriate to describe her as a feminist rationalist. A widespread belief in equal women's rights cannot be observed in the representatives of enlightened rationalism and of Protestantism in general. For them, the story of creation, among other biblical passages, still provided the decisive reason for the subordinate, unequal position of women: Man was created before woman and to have independence; he was master, she helper and unthinkable without him^128^.

Auguste Schmidt, the long-time deputy of Louise Otto-Peters in the ADF, was also influenced in her thoughts on women's emancipation by religious interpretations. This shone through during her speech at the opening of the first German women's conference in Leipzig in 1865. Nevertheless, it is currently an open research question as to what extent specific religious concepts constituted a source of commitment to women's emancipation for Louise Otto-Peters' fellow campaigners in the ADF. This is all the more true since the first phase of the association's development between 1865 and the beginning of the 1890s was marked by the cooperation of Protestants, Jews, and non-denominational women—at least on the board and the committee of the ADF. This is reflected, among other things, in the cooperation of Louise Otto-Peters, Auguste Schmidt, Henriette Goldschmidt and Marianne Menzzer, who were members of the Leipzig board for decades; the former two as Protestants, Henriette Goldschmidt as Jewess and wife of the Rabbi of the Jewish community in Leipzig, and Marianne Menzzer as a supporter of the free churches^129^.

It might be worthwhile to ask when, in which constellations and contexts and in which media these women expressed their specific religious convictions. An initial, though fleeting look at the “Neue Bahnen,” the ADF's fortnightly joint association newspaper, reveals that it relatively rarely contains religious convictions that were formulated as guiding principles. There are contributions on the importance of Martin Luther and the Reformation for the school education of girls, or short reports on women's activities in non-denominational communities. In the latter, the connections of religious and women's emancipation as established by Sylvia Paletschek—albeit for the “Vormärz” era—shine through^130^. The “Kulturkampf” (*cultural struggle*) also finds little expression in “Neue Bahnen.” What expression does exist, is, however, approving in its rejection of the papacy and the Jesuits. Likewise, reports on “agitation against Jews” or Jewish emancipation are marginal.

According to my thesis, religious tolerance and an overarching interdenominational or interreligious consensus that religion was a private matter prevailed in the ADF as shaped by Louise Otto-Peters. This included referring publicly to one's own religious convictions without offending those of other faiths, as Iris Schröder has already established in the case of the ADF of the 1870s^131^. However, activities relating to women's emancipation were clearly at the heart of the association's magazine. Women of different religious, political and social orientations created the ADF as a space for discussing and implementing their own, principally identical interests, because their legal position was principally identical. The ADF provided them with a common umbrella organization for action toward women's emancipation that did not exist in any other context of life for the women involved. This was to change at the end of the nineteenth century, when the churches opened up to discussing certain “women's issues,” and the denominational women's movement came into being.

In the foreword to her 1873 book “Weihe des Lebens” (*Consecration of life*), Louise Otto-Peters stated that she lived in the “trinity of work, endeavor and enthusiasm.” But, for her, “in looking up to God and being steeped in the human task,” lay the glory of life. In more detail, she says:

*“But if you ask me: how and by what means did I keep myself free from destruction and despair, even in the most difficult hours of my life? How did I manage never to tire of working, never to lose heart in my endeavors, never to feel the current of enthusiasm ebb—then I have only one answer: Because I knew that God was and is above me, that I, his child, spirit of his spirit, walk on this earth not only to enjoy its blessings, but to exert all my strengths, as much as possible, to help humankind towards perfection” (Otto*, *1873**)*.

This is followed by commandments and prohibitions in the name of humanity, and chapters on God, reason, nature, humankind, the turn of the year and the guiding stars of life. In the chapter “Die Menschheit” (*Humankind*), she states that every human being carries their individual divinity above, inside and with them; for this reason alone it would be foolish to seek to impose the same faith on all human beings (Otto, 1873, p. 74). In her view, love for humankind meant love for everyone in its ranks, all its strengths and works. “If we love humankind, we will love all human beings, without excepting a single one” (Otto, 1873, p. 77). However, as Louise Otto-Peters emphasized in the foreword that her book provided a free adaptation of didactic pieces by her favorite philosopher K. C. F. Krause, we can ask at this juncture: What is the source of her religious tolerance? Is it the Bible, Krausism, the discourse on fundamental rights of the Enlightenment, the practical cooperation—perceived as necessary—of all women in the ADF interested in emancipation, or is it, somehow, everything together?

## Conclusion and Outlook

If we take a look back at the questions inspiring this article, a first finding is that the feminist aspiration to equal rights for men and women was shared by the women gathering around Louise Otto-Peters in the ADF. Work played a key role in the implementation of their aspiration in three respects: Firstly, as gainful employment in order to have a basis for economic independence in the event of precarious widowhood, employment problems of the husband/father or of being unable or unwilling to marry. To be able to feed oneself—and possibly children, parents etc.—not to be dependent on alms and to be able to determine one's own life to some extent were the imagined goals here. Secondly, associated with work was the idea of the creative development of one's own talents and strengths, of self-realization in the service of humankind. This ought to be work that was useful for society. Self-development and focusing on the public good were inseparably linked here. The work could, but did not have to be paid work; it could also be done in one's own family, for example by bringing up children, or by volunteering, such as in a women's association. This position seems to me to have been widely accepted among the founders of the ADF, in which single, married and widowed women worked together. The areas of society that this self-development in the service of humankind was supposed to take place in depended strongly on the specific understanding of femininity, but also on the political and religious convictions of the individual woman—with Louise Otto-Peters there were no prescribed limits.

Thirdly, work could also be understood as an all-encompassing entitlement of every individual to self-realization and participation in social development toward a better world. It was possible to prove that Louise Otto-Peters had such a comprehensive vision of emancipation, one that was supposed to span class and gender, but was also open in terms of national and religious affiliation. Whether her fellow campaigners shared this vision, or whether there was even a second woman in Germany in 1876 with such a liberal and democratic vision of the future, I am unable to say at present. It also remains an open question whether Louise Otto-Peters developed any ideas about how such a future would have to be shaped socio-economically in terms of ownership structures in order to be able to function, or whether she left it at the stage of an ideal draft plan.

This already says something about how the new relationship between the sexes and the new society were envisaged. The new society needed to differ from the old in varying degrees, depending on whether the primary goals were equal access to employment, fulfillment in professional life and economic independence of women, whether the possible participation of women in all areas of social life was imagined, or whether the free, self-determined participation of all human beings in the service of humanity was conceived of. In any event, society had to change. The first step toward the equal participation of all women in professional life was to achieve the opening up of areas that were previously barred to women, such as the academic professions. This required a fundamental reform of the girls' school system, so that girls could acquire the entrance requirement for studying, the “Abitur.” The same applied to all areas of employment that were hitherto barely accessible to girls and women. However, free access to employment for women also meant the removal of private-law barriers, such as the removal of the tutelage of husbands and fathers over women who were not yet of age. Free development of one's own talents in all areas of private and social life, however, required changes in the entire legal system—not only in education and employment, but also in marriage and family law, and in civic rights. Admittedly, legal equality did not yet mean actual equality; also required was a fundamental change in socialization, customs, traditions and habits in the daily coexistence of the sexes. Comprehensive participation of all human beings in the progress of humankind meant corresponding changes in legal systems and habits for the members of all social classes and strata, ethnic groups, religions, age groups etc.

Regardless of how far emancipation was intended to go, there was agreement on the path of peaceful reform toward Utopia. Starting with oneself and with the mutual help and support of women, the existing society was expected to begin to change. In this process, Louise Otto-Peters and her fellow campaigners understood the civic association to be the decisive organizational means. This also applied to women if, as a disadvantaged social group, they were to become active beyond their own, more or less narrowly drawn personal framework, and if they were not only to introduce their own concerns into the public discussion, but also to ensure these were put into practice. The ADF differed from all other associations in its aspiration to be a women's organization based on solidarity across all classes. The organizational principle of self-help had great consequences for personal development, because the participants acquired self-confidence, assertiveness and, in certain cases, leadership qualities through independently forming opinions and through independent action. This already represented a slice of alternative life in the here and now: They created a specific female association culture that fostered solidarity and friendships between women. This strengthened those prospective forms of life in which individual initiative, sisterhood and solidarity among women played a role.

All in all, an extraordinarily strong influence exerted by the “middle-class system of values” on Louise Otto-Peters and her fellow campaigners becomes apparent. What they devised was an inner- worldly program of how to lead one's life and create meaning, a secular plan for the future. It revolved around middle-class “ultimate” values, such as work, education, independence, self-help and progress, as they were imagined for male ways of life^132^. In the belief in their usefulness for the development and permanent self-improvement of one's personality, the self, in accordance with the progress of humankind, they were truly super-elevated and made sacred (cf. Nipperdey, 1990). All of this was also, to take up an expression of Louise Otto-Peters, something she “believed and recognized herself”^133^. There was, however, one important difference: She saw the guiding stars of middle-class life as instruments in the readjustment of the female life plan. In this respect, her visions represented an alternative middle-class concept of gender and society in competition with dominant middle-class ideas. In it, the equal participation of women was the fundamentally different approach, not contained in the original concepts of almost all master thinkers.

However, the formative influence of the “middle-class system of ideas” on the founders of the ADF also becomes apparent inasmuch as they assumed the dissimilarity of men and women. With their notions of femininity, they tapped into the prevailing gender discourse of difference. In contrast to this discourse, though, they were convinced that the sexes, despite their differently imagined psychological constitution and the resulting different abilities, were of equal value. Depending on how important they as individuals estimated so-called natural psychological differences of the sexes to be, their deliberations differed as to whether there were areas for which women would be more suitable than for others. Some deduced certain specific female character traits from the biological ability to be a mother. In the context of the concept of “organized motherliness,” they considered areas of education, nursing and caring to be particularly suitable, and argued for corresponding professions and voluntary posts to be opened up to women. This is what Henriette Goldschmidt stands for. By contrast, the “eternal feminine,” introduced by Louise Otto-Peters, in its significance for all areas of human existence meant no restrictions for women, corresponding as it did with her comprehensive vision of emancipation. Whether, alongside it, there were also positions in the emerging women's movement—inside or outside the ADF—that did not associate biological difference with psychological difference, but assumed fundamental psychological equality, remains to be investigated further. I have not yet been able to identify them among the persons I am aware of^134^. Ultimately, these always were (and are?!) beliefs, and therefore gender can be imagined as constructed unavailability or as a transcendent resource^135^ to legitimize the desired gender order.

The founders of the ADF used different lines of argumentation and interpretations to promote the all- unifying idea of equal rights for men and women. Characteristic of Louise Otto-Peters is that she was an extraordinarily well-read personality with a wide range of interests, who had acquired a considerable amount of expertise autodidactically. She developed a high degree of critical reflexivity and assessed socio-economic, religious, political and other developments in a clear-sighted manner.

In the lines of argumentation traced here, her examination of the serious technical and socio- economic changes in their effects on family life has been made clear, as has her examination of the so-called natural female gender characteristics, her examination of the human rights discourse of the Enlightenment, and of the religious reform or renewal movement of German Catholicism.

Regarding her essay “Das Recht der Frauen auf Erwerb,” it is possible to identify the interweaving of different lines of argumentation; different interpretations thus had an intensifying effect, so to speak, toward a certain goal. Again and again, the arguments included complementing philosophical and religious interpretations, with some of them being interchangeable. For Louise Otto-Peters, science and religion, knowledge and faith were not strictly separate areas. She regarded the things she believed and the things she knew to be of equal significance; secular and religiously transcendent interpretations could thus be linked. Transcendence of nature became apparent where it tied into natural law and the idea of natural equality and equal human dignity of all human beings. Religious transcendence took place where God created man and woman in different but equal ways. In Louise Otto-Peters, philosophical—i.e., scientific—and religious interpretations met in the assumption that God had given men and women their natural disposition. The spirit of the Enlightenment with its human rights discourse was, in this respect, an advance into the core of Christianity beyond ecclesiastical dogmas, since coming closer to God meant recognition of God.

In addition to those traced here, further lines of argumentation deserve consideration, such as those having recourse to history with regard to personalities, events and developments of significance to women's emancipation, or those referring back to literature, music and art, to developments abroad as well as to modern science and new expert opinions on the nature of the sexes^136^. It is therefore difficult to find an answer as to whether religious interpretations played a special role in legitimizing or transcending Louise Otto-Peters' thinking on the emancipation of women. Against the backdrop of great social change, I would term it a lifelong search for a personal religious worldview in which her specific world of experience as a woman was individually and unmistakably linked to the reception of political, religious, social and other trends prevailing at the time^137^. In this world of ideas, composed of belief and knowledge, only those aspects endured that gave her stability and strength in her striving for a “dignified existence for all, including for women.” It was in this striving that she saw her destiny.

## Author's Note

This is an slightly modified English language translation of Schötz (2014b). Laura Park prepared this translation. Permission was granted by transcript Verlag, Hermannstraße 26, D-33602 Bielefeld; rights@transcript-verlag.de.

## Author Contributions

The author confirms being the sole contributor of this work and has approved it for publication.

### Conflict of Interest Statement

The author declares that the research was conducted in the absence of any commercial or financial relationships that could be construed as a potential conflict of interest.

## Footnotes

^1^Cf. the reproduction of the content of the speech by Otto-Peters (1890). Communicated on the basis of the minutes.

^2^The first time that French women's rights activists used the concept of feminism as a central political idea against what they believed to be the prevailing masculinism of the Third Republic was in the 1880s. In Germany, it was mostly only taken up in the twentieth century, where terms such as “social dimensions of women's issues,” “women's movement” and “women's emancipation” were used for a long time. The specific contents can only be analyzed in historical terms; at the core, however, was or is the rejection of the view of women as a secondary or subordinate group to men, and the resulting unequal rights of participation of women. This often involves the development of strategies and measures to implement better life chances for women, mostly incorporated in more or less comprehensive reforms of society as a whole. Today, a multitude of feminist theories and currents exist (cf., among others, Offen, 2000; Metz-Göckel, 2003; Cudd and Andreasen, 2005; Hark, 2007; Becker-Schmidt and Knapp, 2011; Knapp, 2012).

^3^Regarding the noteworthiness of holding the first German Women's Conference from 15th to 18th October 1865, on the anniversary of the Battle of the Nations in Leipzig, and the founding of the ADF on 18th October resulting from this, on the exact date of the decisive day of the Battle of the Nations, see Schötz (2013b).

^4^Cf. for example, Bussemer (1988, p. 190), Gerhard (1990), and Schaser (2006).

^5^Herrad-Ulrike Bussemer in particular has dealt more intensively with the first decade of the ADF. Cf. H.-U. Bussemer: Frauenbewegung. The most comprehensive depiction of the ADF up to the beginning of the 1890s continues to be the overview by Gerhard (1990). Cf. U. Gerhard: Unerhört (cf. also Twellmann, 1972; Koepcke, 1981; Boetcher and Boetcher, 1983).

^6^Cf. the instructive bibliography in A. Schaser: Frauenbewegung.

^7^Iris Schröder is the only one to have made initial observations on the religious consensus in the ADF in the 1870s, which I will return to later (cf. Schröder, 2001).

^8^The investigations of Sylvia Paletschek, Iris Schröder, and Britta Konz are the main exceptions. The former deals with the connection between religious emancipation, democracy and women's emancipation in the period preceding the March Revolution in 1848, and characterizes the liberal women's associations as pillars of the early German women's movement of the 1840s (cf. Paletschek, 1990). Iris Schröder on the other hand, is interested, among other things, in how religious perceptions and interpretations manifested themselves in the women's movement at the turn of the twentieth century, and in particular to what extent social reformers refer to their religious convictions. Cf. I. Schröder: Arbeiten. Britta Konz also shows that it was possible for social work to have a sound theological basis (cf. Konz, 2004; cf. for the British women's movement, Schwarzkopf, 2011).

^9^Cf. U. Gerhard: Unerhört, section “Auflehnung gegen Gottes Ordnung”? – Die konfessionelle Frauenbewegung, pp. 201–207, and A. Schaser: Frauenbewegung, section IV.3: Die Gründung der konfessionellen Frauenverbände, des Verbandes fortschrittlicher Frauenvereine und des Verbandes Deutscher Hausfrauenvereine, pp. 44–48.

^10^Cf. regarding the spiritual development, in particular, the essays by Johanna Ludwig, Irina Hundt, Susanne Schötz, Barbara Bauer, Siegfried Wollgast, and Ingrid Deich in Louise Otto-Peters Jahrbücher I–III, edited by Johanna Ludwig, Elvira Pradel, and Susanne Schötz in Sax Verlag Beucha, 2004, 2006, 2010. Details are given in the depiction. Regarding the biography of Louise Otto-Peters, see also Ludwig, Johanna: Louise Otto-Peters (pseudonyms: Otto Stern, Malwine von Steinau), in: Sächsische Biografie, published by the Institut für Sächsische Geschichte und Volkskunde e. V., edited by Martina Schattkowsky. Available online at: http://www.isgv.de/saebi/ (accessed August 31, 2018).

^11^See also the Louise-Otto-Peters Archive of the Louise-Otto-Peters-Gesellschaft Leipzig e. V., whose aim is to collect all written material by and about Louise Otto-Peters (cf. for an appraisal, Schötz, 2013a).

^12^Cf. U. Gerhard: Unerhört, pp. 73, 280, and von Gelieu (2008).

^13^Cf. L. Otto-Peters: Vierteljahrhundert, p. 12.

^14^Cf. R.-E. Boetcher Joeres: Anfänge, p. 21.

^15^An appraisal of her concrete practical work cannot be made at this point (cf. Schötz, 2014a).

^16^She wanted to publish the book under the title “Das Recht der Frauen” but could not find a publisher for it. The Hamburg publishing house Hoffmann und Campe finally published it in 1866 under the aforementioned title. See Otto-Peters (1997). Republication of the first edition from 1866, with a reminiscence by the author and reflections on the text from today's perspective.

^17^L. Otto-Peters: Vierteljahrhundert, p. 10.

^18^L. Otto-Peters: Vierteljahrhundert, p. 10.

^19^L. Otto-Peters: Vierteljahrhundert, p. 10.

^20^L. Otto-Peters: Recht, p. 59.

^21^L. Otto: Frauenleben, p. 179.

^22^L. Otto-Peters: Recht, p. 48.

^23^L. Otto: Frauenleben, p. 179.

^24^L. Otto: Frauenleben, p. 171.

^25^L. Otto-Peters: Recht, p. 21.

^26^L. Otto: Frauenleben, p. 179.

^27^Cf. L. Otto-Peters: Recht, in particular the last section “Fortschritte und Aussichten weiblicher Erwerbstätigkeit”, pp. 99–107.

^28^She really does speak of prophecies, cf. L. Otto: Frauenleben, p. 261.

^29^L. Otto: Frauenleben, p. 261.

^30^L. Otto: Frauenleben, p. 263.

^31^L. Otto: Frauenleben, p. 261. Only experiments would be able to decide this question, but these had never been undertaken.

^32^L. Otto: Frauenleben, p. 253.

^33^L. Otto: Frauenleben, p. 256.

^34^L. Otto: Frauenleben, p. 254.

^35^Formerly a technical term of Roman law, the term emancipation became a political term with anti-class content at the end of the eighteenth century, implying that group-specific rights were to be abolished. Around 1830, “emancipation” had become a commonly used catchword.

^36^She repeatedly criticized them, describing their attempt at emancipation as a “hoax.” In her opinion, the barriers crossed by the so-called emancipated of the 1840s concerned only external habits and customs but “not the seriousness of striving and work”. According to Louise Otto-Peters, these emancipated women sought to do the same as men purely in terms of their appearance, sometimes wearing male clothes and in particular, imitating the bad habits of the male sex, for example, by trying to outdo each other in smoking and drinking, spending days and nights in public houses, and so on. Cf. L. Otto-Peters: Recht, p. 48, 67. Irrespective of whether this evaluation does justice to the endeavors of the Saint-Simonians or of someone like Louise Aston, whom Louise Otto-Peters assessed critically on a number of occasions, what does become clearer is her own understanding of women's emancipation, in which the struggle for participation and self-realization as well as cooperation for the sake of the common good were of central importance.

^37^L. Otto: Frauenleben, p. 266.

^38^L. Otto: Frauenleben, p. 258.

^39^L. Otto: Frauenleben, p. 262.

^40^L. Otto: Frauenleben, p. 257.

^41^L. Otto: Frauenleben, p. 257.

^42^L. Otto: Frauenleben, p. 257.

^43^L. Otto-Peters: Recht, p. 71.

^44^L. Otto: Frauenleben, p. 265.

^45^Cf. L. Otto: Frauenleben, p. 261.

^46^Cf. L. Otto: Frauenleben, p. 261, and p. 268.

^47^L. Otto-Peters: Recht, p. 72.

^48^L. Otto-Peters: Recht, p. 71.

^49^L. Otto-Peters: Recht, p. 71 [emphasis in the original].

^50^L. Otto-Peters: Recht, p. 59, 73.

^51^L. Otto-Peters: Recht, p. 59.

^52^L. Otto-Peters: Recht, p. 76 [emphasis in the original].

^53^L. Otto-Peters: Recht, p. 76 [emphasis in the original].

^54^L. Otto-Peters: Recht, p. 82.

^55^L. Otto-Peters: Recht, p. 82.

^56^L. Otto-Peters: Recht, p. 99.

^57^L. Otto-Peters: Recht, p. 96.

^58^L. Otto-Peters: Recht, p. 96.

^59^L. Otto-Peters: Recht, p. 96.

^60^L. Otto-Peters: Vierteljahrhundert, p. VIII.

^61^Quoted from Neue Bahnen 3 (1868), No. 23, p. 182.

^62^L. Otto-Peters: Vierteljahrhundert, p. 11.

^63^Cf. the empirical and systematic overview of the circle of founders of the ADF in S. Schötz: Bausteine.

^64^Cf. L. Otto-Peters: Vierteljahrhundert, p. 11, 14. Henriette Goldschmidt, for example, initially refused to work in an association in which her husband could not also be a member. However, she joined the ADF and the Leipzig Women's Education Association (FBV) in 1866, and was a member of the board of the ADF from 1867. After the death of Louise Otto-Peters, she became its deputy chairwoman in 1895, and Auguste Schmidt took over the chairwomanship (cf. Schmidt, 1895; Fassmann, 1996).

^65^Cf. on the middle-class gender ideal, in particular (Hausen, 1976; Duden, 1977; Gerhard, 1978; Frevert, 1988).

^66^Cf. for a description of the situation in the mid-60s of the nineteenth century, L. Otto-Peters: Recht, chapter II: Die Unzulänglichkeit der gegenwärtigen weiblichen Erwerbszweige, pp. 33–45.

^67^Cf. H.-U. Bussemer: Frauenbewegung.

^68^(cf. Glaser, 1996, p. 301).

^69^Otto-Peters, Louise: Recht, p. 19.

^70^Otto-Peters, Louise: Recht, p. 34.

^71^Cf. Otto-Peters, Louise: Recht, p. 43.

^72^Modern family history research sees this no differently (cf. Mitterauer, 2009; Gestrich, 2010).

^73^Cf. L. Otto-Peters: Recht, p. 56.

^74^L. Otto: Frauenleben, p. 154.

^75^Cf. H.-U. Bussemer: Frauenbewegung, pp. 195 ff. In 1866, Adolf Lette, Chairman of the Prussian Central Association for the Welfare of Working Classes, founded an association in Berlin to promote the employability of the female sex.

^76^(cf. Ludwig, 2003).

^77^Laute aus den “stillen Jahren”. Series of articles, “Den Frauen” by Otto (1995, p. 75).

^78^Cf. L. Otto-Peters: Recht, p. 29.

^79^Cf. H.-U. Bussemer: Frauenbewegung, p. 199.

^80^Cf. the many pieces of evidence in: L. Otto-Peters: Vierteljahrhundert.

^81^Cf. A. Schaser: Geschichte, p. 28 f.; U. Gerhard: Unerhört, p. 124 f., and Kemp (2002). The term “geistige Mütterlichkeit” (*spiritual motherliness*) was used in 1865 for the first time by Henriette Schrader-Breymann, Friedrich Fröbel's niece. Cf. H.-U. Bussemer: Frauenbewegung, p. 204.

^82^(cf. Schötz, 2011), in particular, pp. 360–365.

^83^Quoted from Wollgast (2004, p. 47).

^84^Quoted from Wollgast (2004, p. 47).

^85^According to Wollgast (2004, p. 47).

^86^Cf. S. Schötz: Bausteine, p. 157 f., sowie Wollgast (2004).

^87^Cf. Neue Bahnen 4 (1869), No. 6, p. 48.

^88^Cf. S. Wollgast: Louise Otto-Peters, p. 45.

^89^This is the view of Yamada (2006, p. 109).

^90^Cf. L. Otto-Peters: Recht, p. 80.

^91^Cf. L. Otto-Peters: Recht, p. 72.

^92^This is how Barbara Greven-Aschoff interprets the “Ewig-Weibliche” in Louise Otto-Peters (cf. Greven-Aschoff, 1981).

^93^This is the view of Diethe (2002).

^94^Cf. B. Greven-Aschoff: Frauenbewegung, p. 37.

^95^Cf. U. Gerhardt: Frauenbewegung, p. 8.

^96^U. Frevert: Meisterdenker, p. 20.

^97^L. Otto-Peters: Vierteljahrhundert, p. 8 [emphasis in the original].

^98^L. Otto-Peters: Recht, p. 99 [emphasis in the original].

^99^L. Otto-Peters: Recht, p. 77.

^100^L. Otto-Peters: Vierteljahrhundert, p. 24.

^101^L. Otto-Peters: Vierteljahrhundert, p. 24.

^102^L. Otto-Peters: Recht, p. 71.

^103^U. Gerhardt: Unerhört, p. 16.

^104^She was probably the first woman to do this publicly in Germany. Cf. Ludwig, Johanna: Rechte, p. 504.

^105^Otto-Peters: Recht, p. 119 [emphasis in the original].

^106^Several clues to her religiosity can be found in Otto (1995), Deich (2004, p. 65–68), and S. Wollgast: Louise Otto-Peters.

^107^L. Otto-Peters: Recht, p. 76.

^108^L. Otto-Peters: Recht, p. 76.

^109^L. Otto-Peters: Recht, p. 71.

^110^L. Otto-Peters: Recht, p. 71.

^111^L. Otto-Peters: Recht, p. 72.

^112^L. Otto-Peters: Recht, p. 72 [emphasis in the original].

^113^Cf. regarding such arguments (Lundt, 1996).

^114^Cf. L. Hölscher: Religiosität, p. 209.

^115^Cf. U. Baumann: Protestantismus, pp. 20.

^116^Cf. I. Hundt: Studien.

^117^Cf. I. Hundt: Studien, p. 35.

^118^Quoted from Ludwig, Johanna: Eigener Wille und eigene Kraft. Der Lebensweg von Louise Otto-Peters bis zur Gründung des Allgemeinen deutschen Frauenvereins 1865. Nach Selbstzeugnissen und Dokumenten, p. 26 [in print, emphasis in the original]. My thanks to the author for the opportunity to see the manuscript.

^119^Otto (1845, p. 1057). In 1846, she met Johannes Ronge in person in Breslau (cf. R.-E. Boetcher-Joeres: Anfänge, p. 54; Otto, 1847).

^120^L. Otto: Wort, p. 1056.

^121^L. Otto: Wort, p. 1056

^122^L. Otto: Wort, p. 1057.

^123^In the article, she called for the emancipation of the Jews (cf. Otto, 1848, p. 61).

^124^These included Robert Blum, Emil Adolph Roßmäßler, Franz Wigard, and Auguste Scheibe.

^125^Among other things, she used the parable of “Martha and Mary”, Luke 10, 38–42; the staff of the “Frauen-Zeitung” were supposed to see themselves as successors of that noble Virgin from Bethany, according to the wish of Louise Otto (cf. Otto, 1849a).

^126^Cf. S. Wollgast: Louise Otto-Peters, p. 57.

^127^Cf. L. Hölscher: Religiosität, p. 210.

^128^Cf. U. Baumann: Protestantismus, pp. 56–66.

^129^Cf. regarding the biography of Auguste Schmidt (Ludwig et al., 2003); on Marianne Menzzer (Stolze, 2005); as well as Scholz (2008); on Henriette Goldschmidt (Kemp and Kemp, 2012).

^130^Cf. S. Paletschek: Frauen.

^131^Cf. I. Schröder: Arbeiten.

^132^Hettling and Hoffmann (2000). Cf. in particular, the introduction by the editors and the essays by Hettling (2000, p. 15) and Kessel (2000, p. 141).

^133^Cf. S. Wollgast: Louise Otto-Peters, p. 45.

^134^Herrad-Ulrike Bussemer considers such egalitarian positions as being dominant in the ADF before 1870, and after that, the concept of “spiritual motherliness.” It is unclear to me who is to be regarded as the representative of the first position. Cf. H.-U. Bussemer: Frauenbewegung.

^135^I am using terminology here that Nele Schneidereit has introduced into the discussion (see also Scherer, 2013).

^136^Cf. here, such lines of argumentation and interpretations in the struggle for the opening of universities and the introduction of studying for women in Schötz, Susanne: Gehirn.

^137^She had this in common with many other members of the educated classes of the nineteenth century. Cf. L. Hölscher: Religiosität, p. 194.

## References

[B1] Bauer G. (2006). Kulturprotestantismus und frühe bürgerliche Frauenbewegung in Deutschland. Agnes von Zahn-Harnack (1884–1950). Leipzig: Evangelische Verlagsanstalt.

[B2] Baumann U. (1992). Protestantismus und Frauenemanzipation in Deutschland 1850 bis 1920. Frankfurt; New York, NY: Campus-Verlag.

[B3] Becker-Schmidt R. Knapp G.-A. (2011). Feministische Theorien zur Einführung. Hamburg: Junius.

[B4] Blaschke O. Kuhlemann F.-M. (eds.). (1996). Religion im Kaiserreich. Milieus – Mentalitäten – Krisen. Gütersloh: Kaiser; Gütersloher Verlagshaus.

[B5] Bock G. (2000). Frauen in der europäischen Geschichte. Vom Mittelalter bis zur Gegenwart. Munich: Beck, 127.

[B6] Boetcher J. Boetcher R.-E. (1983). Die Anfänge der deutschen Frauenbewegung: Louise Otto-Peters. Frankfurt: Fischer Taschenbuch Verlag.

[B7] Breuer G. (1998). Frauenbewegung im Katholizismus. Der Katholische Frauenbund 1903–1918. Frankfurt; New York, NY: Campus-Verlag.

[B8] Bussemer H.-U. (1988). Bürgerliche Frauenbewegung und männliches Bildungsbürgertum 1860–1880, in Bürgerinnen und Bürger. Geschlechterverhältnisse im 19. Jahrhundert, ed Frevert U. (Göttingen: Vandenhoeck und Ruprecht), 190–205.

[B9] Cudd A. Andreasen R. (2005): Feminist Theory: A Philosophical Anthology. Oxford: Blackwell.

[B10] Deich I. (2004). Annäherungen an Louise Otto-Peters' Buch ‘Genius der Natur', in Louise-Otto-Peters-Jahrbuch I, eds Ludwig J. Pradel E. Schötz S. (Beucha: Sax-Verlag), 58–75.

[B11] Diethe C. (2002). The life and work of Germany's founding feminist Louise Otto-Peters (1819–1895). New York, NY: Edwin Mellen Press, 4.

[B12] Duden B. (1977). Das schöne Eigentum. Zur Herausbildung des bürgerlichen Frauenbildes an der Wende vom 18. zum 19. Jahrhundert. Kursbuch 48, 125–140.

[B13] Fassmann I. M. (1996). Jüdinnen in der deutschen Frauenbewegung 1865–1919. Hildesheim; Zürich; New York, NY: Olms, 164.

[B14] Frevert U. (1988). Bürgerliche Meisterdenker und das Geschlechterverhältnis. Konzepte, Erfahrungen, Visionen an der Wende vom 18. zum 19. Jahrhundert, in Bürgerinnen und Bürger. Geschlechterverhältnisse im 19. Jahrhundert, ed Frevert U. (Göttingen: Vandenhoeck und Ruprecht), 17–48.

[B15] Gerhard U. (1978). Verhältnisse und Verhinderungen. Frauenarbeit, Familie und Recht der Frauen im 19. Jahrhundert. Frankfurt: Suhrkamp.

[B16] Gerhard U. (1990). Unerhört. Die Geschichte der deutschen Frauenbewegung. Reinbek bei Hamburg: Rowohlt, 76.

[B17] Gestrich A. (2010). Geschichte der Familie im 19. und 20. Jahrhundert. München: Oldenbourg.

[B18] Glaser E. (1996). ‘Sind Frauen studierfähig?' Vorurteile gegen das Frauenstudium, in Geschichte der Mädchen- und Frauenbildung, Vol. 2: Vom Vormärz bis zur Gegenwart, eds Kleinau E. Opitz C. (Frankfurt; New York, NY: Campus-Verlag), 299–309.

[B19] Götz von Olenhusen I. (ed.). (1995). Frauen unter dem Patriarchat der Kirchen: Katholikinnen und Protestantinnen im 19. und 20. Jahrhundert. Stuttgart; Berlin; Cologne: Kohlhammer.

[B20] Grass K. M. Koselleck R. (1975). Emanzipation, in Geschichtliche Grundbegriffe. Historisches Lexikon zur politisch-sozialen Sprache in Deutschland, Vol. 2, eds Brunner O. Conze W. Koselleck R. (Stuttgart: Klett-Cotta), 153–197.

[B21] Greven-Aschoff B. (1981). Die bürgerliche Frauenbewegung in Deutschland 1894–1933. Göttingen: Vandenhoeck und Ruprecht, 40.

[B22] Hark S. (2007). Dis/Kontinuitäten: Feministische Theorie. Wiesbaden: VS Verlag für Sozialwissenschaften.

[B23] Hausen K. (1976). Die Polarisierung der ‘Geschlechtscharaktere'. Eine Spiegelung der Dissoziation von Erwerbs- und Familienleben, in Sozialgeschichte der Familie in der Neuzeit Europas, ed Conze W. (Stuttgart: Klett), 363–393.

[B24] Hettling M. (2000). Die persönliche Selbständigkeit. Der archimedische Punkt bürgerlicher Lebensführung, in Der bürgerliche Wertehimmel. Innenansichten des 19. Jahrhunderts, eds Hettling M. Hoffmann S.-L. (Göttingen: Vandenhoeck und Ruprecht), 57–78.

[B25] Hettling M. Hoffmann S.-L. (eds.). (2000). Der bürgerliche Wertehimmel. Innenansichten des 19. Jahrhunderts. Göttingen: Vandenhoeck und Ruprecht.

[B26] Hölscher L. (1994). Bürgerliche Religiosität im protestantischen Deutschland des 19. Jahrhunderts, in Religion und Gesellschaft im 19. Jahrhundert, ed Schieder W. (Stuttgart: Klett-Cotta), 191–215.

[B27] Honegger C. (1991). Die Ordnung der Geschlechter. Die Wissenschaft vom Menschen und das Weib 1750–1850. Frankfurt; New York, NY: Campus-Verlag.

[B28] Hübinger G. (1994). Kulturprotestantismus und Politik. Zum Verhältnis von Liberalismus und Protestantismus im wilhelminischen Deutschland. Tübingen: Mohr.

[B29] Hundt I. (2004). Die autodidaktischen Studien ‘eines deutschen Mädchens' um 1840, in Louise-Otto-Peters- Jahrbuch I, eds Ludwig J. Pradel E. Schötz S. (Beucha: Sax-Verlag), 29–38.

[B30] Hundt I. (2006). Eine ‘wahre' Sozialistin? Louise Otto und ihre österreichischen Freunde im Vormärz, in Louise- Otto-Peters-Jahrbuch II, eds Ludwig J. Schötz S. Rothenburg H. (Beucha: Sax-Verlag), 115–133.

[B31] Kaplan M. A. (1981). Die jüdische Frauenbewegung in Deutschland. Organisation und Ziele des Jüdischen Frauenbundes 1904–1938. Hamburg: Christians.

[B32] Kaufmann D. (1988). Frauen zwischen Aufbruch und Reaktion. Protestantische Frauenbewegung in der ersten Hälfte des 20. Jahrhunderts. München: Piper.

[B33] Kemp A. (2002). ‘Wir haben Väter der Stadt, wo bleiben die Mütter?' On the work of Henriette Goldschmidt, in Bildung, Studium und Erwerbstätigkeit von Frauen in Leipzig im 19. und 20. Jahrhundert, ed Moderow H.-M. (Beucha: Sax-Verlag), 63–74.

[B34] Kemp A. Kemp H. (2012). Henriette Goldschmidt – ein Glücksfall für Leipzig, in Henriette Goldschmidt und die Hochschule für Frauen zu Leipzig. Berichte vom 19. Louise-Otto-Peters-Tag 2011. LOUISEum 32. Sammlungen und Veröffentlichungen der Louise- Otto-Peters-Gesellschaft e. V. Leipzig, eds Ludwig J. Kämmerer G. Schötz S. (Leipzig: J. Ludwig), 8–29.

[B35] Kessel M. (2000). ‘Der Ehrgeiz setzte mir heute wieder zu …'. Geduld und Ungeduld im 19. Jahrhundert, in Der bürgerliche Wertehimmel. Innenansichten des 19. Jahrhunderts, eds Hettling M. Hoffmann S.-L. (Göttingen: Vandenhoeck und Ruprecht), 129–148.

[B36] Knapp G.-A. (2012). Im Widerstreit. Feministische Theorie in Bewegung. Wiesbaden: VS Verlag für Sozialwissenschaften.

[B37] Koepcke C. (1981). Geschichte der deutschen Frauenbewegung: von deutschen Anfängen bis 1945. Freiburg im Breisgau; Basel; Vienna: Herder.

[B38] Konz B. (2004). Religion, Emanzipation und gesellschaftliche Verantwortung: Leben und Werk der jüdischen Frauenrechtlerin Bertha Pappenheim, in Vision und Verantwortung. Festschrift für Ilse Meseberg-Haubold, eds Konz B. Link-Wieczorek U. (Münster: Lit-Verlag), 38–52.

[B39] Ludwig J. (2003). ‘Auch die Rechte der Frauen bedenken'. Louise Otto (1819–1895) in der Revolution von 1848/49, in Akteure eines Umbruchs. Männer und Frauen der Revolution von 1848/49, eds Bleiber H. Schmidt W. Schötz S. (Berlin: Fides), 493–514.

[B40] Ludwig J. Nagelschmidt I. Schötz S. Berndt S. (eds.). (2003). Leben ist Streben. Das erste Auguste-Schmidt-Buch. Reden, Vorträge und Dokumente der Ehrungen zum 100. Todestag der Pädagogin, Publizistin und Frauenrechtlerin Auguste Schmidt am 10./11. Juni 2002 in Leipzig. Leipzig: Leipziger Universitätsverlag.

[B41] Lundt B. (1996). Zur Entstehung der Universität als Männerwelt, in Geschichte der Mädchen-und Frauenbildung. Vol. 1: Vom Mittelalter bis zur Aufklärung, eds Kleinau E. Opitz C. (Frankfurt; New York, NY: Campus-Verlag), 103–118.

[B42] Maierhof G. Wenzel C. (2004). Jüdisch-sein, Frau-sein, Bund-sein. Der jüdische Frauenbund 1904–2004. Ariadne, 45–46.

[B43] Meiwes R. (2000). “Arbeiterinnen des Herrn” Katholische Frauenkongregationen im 19. Jahrhundert. Frankfurt; New York, NY: Campus-Verlag.

[B44] Metz-Göckel S. (2003). Feminismus, in Historisch-kritisches Wörterbuch des Feminismus, ed Haug F. (Hamburg: Argument-Verlag), 170–179.

[B45] Mitterauer M. (2009). Sozialgeschichte der Familie. Kulturvergleich und Entwicklungsperspektiven. Wien: Braumüller.

[B46] Nipperdey T. (1990). Deutsche Geschichte 1866–1918, Vol. 1. München: Beck, 521.

[B47] Offen K. (2000). European Feminisms, 1700–1950: A Political History. Stanford: Stanford University Press.

[B48] Otto C. (1995). Variationen des “poetischen Tendenzromans”. Das Erzählwerk von Louise Otto-Peters. Pfaffenweiler: Centaurus-Verlagsgesellschaft, 266.

[B49] Otto L. (1845). Auch ein Wort, über den 30. November in Dresden. Wandelstern 1054–1058.

[B50] Otto L. (1847). Die Theilnahme der weiblichen Welt am Staatsleben, in Volkstaschenbuch Vorwärts, ed Blum R. (Leipzig: Friese), 46.

[B51] Otto L. (1848). Zur Judenfrage. Ein Wort zur Versöhnung. Der Volksfreund. Sächsische Blätter für alle Interessen des Volkes 59–61.

[B52] Otto L. (1849a). Programm. Frauen-Zeitung 2.

[B53] Otto L. (1849b). Die Freiheit ist unteilbar. Frauen-Zeitung 2.

[B54] Otto L. (1871). Erinnerungsbilder eines deutschen Frauenlebens, X. Die erste deutsche Frauen-Zeitung. Politische Frauen-Zeitung 604.

[B55] Otto L. (1873). Weihe des Lebens. Ein Buch zur Erhebung und Erbauung des Geistes und Herzens. Leipzig: Schäfer, 3.

[B56] Otto L. (1876). Frauenleben im deutschen Reich. Erinnerungen aus der Vergangenheit mit Hinweis auf Gegenwart und Zukunft. Leipzig: Schäfer, 8.

[B57] Otto L. (1995). ‘Den Frauen' in ‘Leipziger Sonntagsblatt', reprinted, in Louise Otto-Peters. Ihr literarisch-publizistisches Werk. Exhibition Catalog, eds. Ludwig J. Jorek R. (Leipzig: Leipziger Universitätsverlag), 65–75.

[B58] Otto-Peters L. (1868). Menschenwürdiges Dasein für Alle! Neue Bahnen 3:122.

[B59] Otto-Peters L. (1890). Das erste Vierteljahrhundert des Allgemeinen deutschen Frauenvereins gegründet am 18. October 1865 in Leipzig. Leipzig: Schäfer, 8 pp.

[B60] Otto-Peters L. (1893). Bücherschau. Neue Bahnen 28:159.

[B61] Otto-Peters L. (1997). Das Recht der Frauen in: Otto-Peters, Louise: Das Recht der Frauen auf Erwerb in LOUISEum 7. Sammlungen und Veröffentlichungen der Louise-Otto-Peters-Gesellschaft e. V., eds Franzke A. Ludwig J. Notz G. Louise-Otto-Peters-Gesellschaft e V. Götze R. (Leipzig: Leipziger Universitätsverlag), 10.

[B62] Paletschek S. (1990). Frauen und Dissens. Frauen im Deutschkatholizismus und in den freien Gemeinden 1841–1852. Göttingen: Vandenhoeck und Ruprecht.

[B63] Schaser A. (2006). Frauenbewegung in Deutschland 1848–1933. Darmstadt: Wissenschaftliche Buchgesellschaft, 41.

[B64] Scherer A. (2013). Conference report on ‘Das Geschlecht der Transzendenz – Bilder, Narrative, Werte'. Dresden, in H-Soz-u-Kult. Available online at: http://hsozkult.geschichte.hu-berlin.de/tagungsberichte/id=4651 (accessed August 31, 2018).

[B65] Schmidt A. (1895). Henriette Goldschmidt. Neue Bahnen 30, 185–187.

[B66] Scholz S. (2008). Marianne Menzzer – Ein biografisches Porträt (Unpublished Bachelor's thesis in History). Technische Universität Dresden, Dresden, Germany.

[B67] Schötz S. (2005). Von 1848 nach 1865? Bausteine zu einer Kollektivbiographie der Gründerinnen und Gründer der deutschen Frauenbewegung, in Revolution und Reform in Deutschland im 19. und 20. Jahrhundert. First half-volume: Ereignisse und Prozesse. Zum 75. Geburtstag von Walter Schmidt, eds Bleiber H. Küttler W. (Berlin: Trafo-Verlag), 151–164.

[B68] Schötz S. (2011). ‘Gleiches Gehirn, gleiche Seele, gleiche Rechte!' Der Allgemeine Deutsche Frauenverein im Ringen um die Öffnung der Universitäten für Frauen, 1865 bis 1890, in Schule in Leipzig. Aspekte einer achthundertjährigen Geschichte, eds Döring D. Flöter J. (Leipzig: Leipziger Universitätsverlag), 347–373.

[B69] Schötz S. (2013a). 20 Jahre Louise-Otto-Peters-Gesellschaft in Leipzig. In Dankbarkeit gewidmet Johanna Ludwig (January 26th, 1937–August 2nd, 2013). JahrBuch für Forschungen zur Geschichte der Arbeiterbewegung 12, 157–168.

[B70] Schötz S. (2013b). Frauenschlacht zu Leipzig. Anmerkungen zu Louise Otto-Peters in der Reichsgründungszeit, in Helden nach Maß 200 Jahre Völkerschlacht bei Leipzig, ed Rodekamp V. (Leipzig: Stadtgeschichtliches Museum Leipzig), 47–54.

[B71] Schötz S. (2014a). Leipzig und die erste deutsche Frauenbewegung, in Leipzigs Bedeutung für die Geschichte Sachsens, ed Döring D. (Leipzig: Leipziger Universitätsverlag).

[B72] Schötz S. (2014b). Menschen werden wollen die Frauen und teilnehmen am Kranz der Arbeit und des Sieges. Visionen von Emanzipation, Gemeinsinn und Gesellschaftsreform in der ersten deutschen Frauenbewegung, in Wirtschaft und Gemeinschaft. Konfessionelle und neureligiöse Gemeinsinnsmodelle im 19. und 20. Jahrhundert, eds Steinberg S. Müller W. (Bielefeld: Transcript-Verlag), 171–215.

[B73] Schröder I. (2001). Arbeiten für eine bessere Welt. Frauenbewegung und Sozialreform 1890–1914. Frankfurt: Campus-Verlag, 166–171.

[B74] Schwarzkopf J. (2011). Women's mission. Die Bedeutung von Religion in der ersten britischen Frauenbewegung bis 1914. Ariadne 60, 36–41.

[B75] Stolze E. (2005). Ins Licht gerückt: Marianne Menzzer (27. 11. 1814–5. 6. 1895), Ehrenmitglied des Allgemeinen deutschen Frauenvereins, in Auf den Spuren frauenbewegter Frauen. Berichte vom 12. Louise-Otto-Peters-Tag 2004. LOUISEum 23. Sammlungen und Veröffentlichungen der Louise-Otto-Peters-Gesellschaft e. V. Leipzig, ed Ludwig J. (Leipzig: Ludwig), 25–30.

[B76] Twellmann M. (1972). Die deutsche Frauenbewegung. Ihre Anfänge und erste Entwicklung 1843–1889. Meisenheim am Glan: Verlag Anton Hain.

[B77] von Gelieu C. (2008). Vom Politikverbot ins Kanzleramt – Ein hürdenreicher Weg für Frauen. Berlin: Lehmanns Media.

[B78] Wenzel C. Wolff K. (1999). Im Namen des Herrn? Konfessionelle Frauenverbände 1890–1933. Ariadne 35:72.

[B79] Wollgast S. (2004). Louise Otto-Peters und Karl Christian Friedrich Krause als ihre philosophische Quelle, in Louise-Otto-Peters-Jahrbuch I, eds Ludwig J. Pradel E. Schötz S. (Beucha: Sax-Verlag), 39–57.

[B80] Yamada T. (2006). Louise Otto-Peters und die deutschkatholische Bewegung. Die bürgerliche Frauenbewegung des Vormärz und der Revolutionszeit, in Louise-Otto-Peters-Jahrbuch II, eds Ludwig J. Schötz S. Rothenburg H. (Beucha: Sax-Verlag), 90–114.

